# Experiences of loneliness among people with a diagnosis of ‘personality disorder’: A qualitative interview study in the UK

**DOI:** 10.1371/journal.pmen.0000671

**Published:** 2026-08-10

**Authors:** Sarah Ikhtabi, Nicola Morant, Annabel Rushforth, Sarah Rowe, Eiluned Pearce, Oliver Dale, Alexandra Pitman, Sonia Johnson

**Affiliations:** 1 Division of Psychiatry, University College London, London, United Kingdom; 2 Clinical and Applied Psychology Unit, University of Sheffield, Sheffield, United Kingdom; 3 Department of Experimental Psychology, University of Oxford, Oxford, United Kingdom; 4 Sussex Partnership NHS Foundation Trust, Worthing, United Kingdom; 5 North London NHS Foundation Trust, London, United Kingdom; University of Turin, ITALY

## Abstract

Loneliness is common among people diagnosed with a ‘personality disorder’ and addressing this is a recovery goal. Little qualitative research has explored the experience of loneliness among people with a diagnosis of ‘personality disorder’. This study aimed to provide an in-depth understanding of loneliness among people with a ‘personality disorder’ diagnosis, and approaches used to alleviate it, informing future psychosocial intervention co-production. We conducted semi-structured interviews with 16 people in the United Kingdom from a range of sociodemographic backgrounds via charities and research networks. Data were analysed using reflexive thematic analysis. We identified five overarching themes: 1) the experience of loneliness, 2) the mutually reinforcing relationship between loneliness and mental health difficulties, 3) the perceived origins of loneliness, 4) a unique form of stigma, and 5) recovery rooted in managing to find a sense of belonging. Our findings suggest that loneliness is a painful and enduring experience that participants perceived to originate from invalidating early childhood experiences. These early experiences contributed to ongoing feelings of loneliness, persisting into adulthood. Loneliness was perceived to compound features of ‘personality disorder’ and *vice versa*. Experiences of stigma and discrimination related to the diagnosis of ‘personality disorder’ and any protected characteristics presented another barrier to achieving connections with others, further playing into an apparent mutually reinforcing relationship between loneliness and features of ‘personality disorder’. Our findings indicate a clear need to address loneliness in this group. The findings help identify potential targets for psychosocial interventions aimed at reducing loneliness and promoting social connectedness in people with a diagnosis of ‘personality disorder’. Psychosocial interventions, combining valued group-based activities and/or safe social relationships alongside developing psychological skills for self-understanding and acceptance, have the potential to reduce feelings of loneliness.

## Introduction

There is rising interest in social factors associated with mental ill health among people with a diagnosis of ‘personality disorder’ [1,2]. Recent systematic reviews of the quantitative literature [2,3] and a meta-synthesis of the qualitative literature [1] show that people with a diagnosis of ‘personality disorder’ experience an intense sense of disconnection and high levels of loneliness. However, loneliness has tended to be overlooked as a potential intervention target and there is a lack of research focused on understanding loneliness and its perceived links to mental health among people with a diagnosis of ‘personality disorder’ [1,2,4]. Identifying the ways people with a diagnosis of ‘personality disorder’ experience and cope with loneliness can shed light on strategies that could be incorporated into an intervention tailored to their social needs.

‘Borderline personality disorder’, also known as ‘emotionally unstable personality disorder’ (EUPD) in the International Classification of Disease (ICD), is the most prevalent and frequently encountered condition in clinical practice [5,6]. People diagnosed with ‘personality disorder’ particularly ‘EUPD’ often face exclusion and stigma from the general public and within mental health services [7]. This is driven by the negative connotations associated with the condition and misconceptions around treatability [8,9]. Throughout this paper, we use quotation marks and the phrase “diagnosed with a ‘personality disorder’”, as suggested by people with lived experience of being diagnosed with ‘personality disorder’ [10] to acknowledge the strong critiques regarding the diagnostic label which include the inherently invalidating nature of the term [8]. We also use the language and labels used by participants in the interview study to capture their perceptions and preferred labels (e.g., ‘complex emotional needs’).

Loneliness is a painful and distressing emotional state characterised by a perceived discrepancy between one’s actual and desired quantity and/or quality of social relationships [11]. Loneliness is more prevalent among people with mental health problems compared to the general population and is linked to a wide range of mental health and physical health problems [12,13]. A recent umbrella review exploring the association between social constructs, including loneliness, and mental health conditions found that loneliness is associated with severity of mental health symptoms and a range of conditions including post-traumatic stress disorder, eating disorders, anxiety, depression and psychosis [13].

Our previous meta-synthesis of the qualitative literature exploring experiences of loneliness among people with a diagnosis/traits of ‘personality disorder’ suggests that loneliness, difficulties forming satisfying social connections, and a lack of a sense of belonging are common and painful experiences for this group, potentially rooted in early alienating and invalidating childhood experiences [1]. This is important because the evidence suggests that a sense of safety, often achieved through a sense of belonging and social connectedness, is crucial in crisis management and promotes both symptomatic reductions and personal recovery outcomes [1,14]. Considering the painful nature of loneliness and the impact of loneliness on both symptomatic recovery and personal recovery, understanding and tackling loneliness could be a promising intervention target for people with a diagnosis of ‘personality disorder’ [15,16].

Our previous meta-synthesis also showed consistent weaknesses across qualitative studies exploring loneliness-related experiences among people with a diagnosis/traits of ‘personality disorder’. Some of these limitations included lack of reflexivity and lived experience involvement, and lack of diversity or representative samples [1]. Additionally, only two qualitative studies (conducted by the same author) specifically explored loneliness among people with a diagnosis of ‘personality disorder’ as opposed to only passing references to it [17,18]. These limitations are important to address in future studies, particularly given the lack of progress in research focusing on delivering good care specifically to people with a diagnosis of ‘personality disorder’ [4,19] and the narrow focus on reduction of suicidality despite evidence indicating that social and relational challenges may often precede suicidality [20,21]. This suggests that interventions may be lacking a focus on other recovery outcomes highly valued by people with a diagnosis of ‘personality disorder’ [15,22].

Given the above limitations in qualitative studies and the high prevalence of loneliness reported by people with a diagnosis of ‘personality disorder’, there is a clear need for co-designed qualitative research exploring experiences of loneliness and potential acceptable social and psychological approaches to reducing loneliness in this group. There is also a need to include the perspectives of people from a range of ethnicities, sexualities, age groups, and genders.

We therefore aimed to conduct a qualitative interview study to address the following research questions:

1) How is loneliness perceived and described by people diagnosed with ‘personality disorder’ and what are the reported links between loneliness and ‘personality disorder’ and mental health and recovery among people with a diagnosis of ‘personality disorder’ in the United Kingdom (UK)?
2) What are the perceived origins of loneliness among people with a diagnosis of ‘personality disorder’?
3) What factors might alleviate or worsen loneliness experiences in people with a diagnosis of ‘personality disorder’?
4) How can awareness of these factors be used to develop acceptable psychological and social approaches to reduce feelings of loneliness in this group?

## Methods

We used a qualitative exploratory approach to conduct an in-depth semi-structured interview study, as a collaboration between experts by lived experience, clinicians, and researchers. We took a critical realist approach which is well suited to studies exploring complex phenomena and experiences shaped by social structures [23]. This captures a “multilayered social reality” [24,25] and acknowledges both an observable reality and subjective interpretations shaped by social structures, interactions and feelings [24–26]. In line with this, we used latent coding where analysis went beyond descriptive surface level content to identify underlying meaning [27,28]. As deductive and inductive approaches lie on a continuum [28], we used a more inductive approach in this study. We used the Consolidation Criteria for Reporting Qualitative Research (COREQ) to guide reporting of methods and findings [29].

### Ethics statement

Ethical approval was obtained from University College London Research Ethics Committee (ref: 23645/001). At the start of the interview, the primary researcher (SI) rechecked participants’ capacity to provide informed consent and obtained verbal consent conducted remotely via Microsoft Teams. This involved asking participants questions about the study and asking to repeat back key points in order to assess understanding, retention, weighting of information and communications. A follow-up email/phone call (within three days) was offered to reflect on the interview and offer signposting.

### Research team

The wider research team included those with relevant lived experience and clinical and/or research experience of loneliness and ‘personality disorder’. The authorship team consisted of eight individuals: two female lived experience researchers (AR, SI), five female clinical and non-clinical academics working in the field of loneliness and mental health, one of whom is a qualitative research expert (NM), and one male clinician specialising in ‘personality disorder’ (OD); all were from White backgrounds and European descent except for SI who is from an Arab background. The authorship team were involved in developing the research questions, designing the topic guide and recruitment plan, analysing a proportion of interview transcripts, refining the thematic framework, and interpreting the findings. The primary researcher (SI) who conducted all the interviews and led analysis has experiential knowledge of and research experience in the topic of loneliness and ‘personality disorder’. In addition to the authorship team, two lived experience advisors (one male, one female), who elected not to be part of the authorship team, were involved in developing the research questions, topic guide, and recruitment plan, providing advice on ethical decisions, coding a proportion of interview transcripts, and discussing the thematic framework and findings.

The multidisciplinary nature of our research team ensured that our research questions and topic guide were based on real world lived experiences and allowed for a range of perspectives and helped generate richer and nuanced understanding of data which enhanced reflexivity and validity of findings.

### Recruitment

We recruited adults (18 years +) who reported that they were lonely and either self-identified as having a ‘personality disorder’ (whether or not they had a formal diagnosis) or were formally clinically diagnosed with a ‘personality disorder. Participants must be residing in the UK, English language speaker, and had no reported cognitive impairment. We included people with psychiatric/physical comorbidities to reflect the clinical reality of living with a ‘personality disorder’ [30,31]. We included self-identification with a diagnosis of ‘personality disorder’ to ensure that people’s own perception of their mental health was included as recruitment was conducted through UK-based ‘personality disorder’ and general mental health charities which may support people who do not have a formal diagnosis. We also recruited via our research networks which include many who participate in research from a lived experience perspective. From the people who expressed an interest in participating, we used purposive sampling to select a diverse sample that included people from a wide range of ethnicities, age groups, sexual orientations, and genders [32]. During recruitment we used strategies such as recruiting through charities/networks that support people from minority groups and changing the advertised recruitment flyer to target specific demographic groups (e.g., ‘people from sexual minorities needed’) to ensure we accessed this range of diversity criteria. Based on existing recommendations in qualitative methods about necessary sample size for interview studies utilising thematic analysis, a sample of 12–20 participants was thought to be sufficient for generating rich data [33,34]. Data collection stopped after 16 interviews were conducted as no new themes were identified at which point the researcher made a judgement to conclude data collection.

### Procedure

Topic guide questions were informed by the literature on loneliness and ‘personality disorder’ and were phrased carefully to allow participants to express their experiences while minimising the potential for suggestion (See S1 Appendix). The topic guide was revised iteratively, and, after a total of two pilot interviews, we added a question to the demographic section on area/location in which the participant resided.

Interested participants were directed to the study website, which provided more information. Participants contacted the lead researcher via email, who checked eligibility, responded to any queries, and provided a Qualtrics form to collect sociodemographic information and elicit signed informed consent via an online form (See S2 Appendix and S3 Appendix). Interviews were conducted online via Microsoft Teams application [35]. One participant dropped out early in the interview for personal reasons and their data were excluded from the analysis. All interviews took place from 10^th^ of February 2023 to 9^th^ of September 2023.

The mean length of interviews was 1 hour and 2 minutes (range: 40 minutes-82 minutes). The online interview was audio recorded with the participant’s consent and then transcribed verbatim and anonymised by SI. Recorded interviews and transcriptions were saved and stored in a separate secure folder.

### Analysis

We used reflexive thematic analysis [27] supported by computer software NVivo [36] with analysis occurring in parallel with conducting interviews. Reflexive thematic analysis is well suited for this study as it enables the researcher to develop an in-depth and nuanced understanding of the data whilst following a well-defined framework [28,37].

We used a broadly inductive approach and followed Braun and Clarke’s six-stage model [28], as follows: 1) Familiarisation with data, 2) Generating initial codes and themes, 3) Searching for themes and meanings, 4) Reviewing and expanding on potential themes, 5) Naming themes, and 6) Reporting themes.

SI was the primary analyst and drew on the multiple perspectives of team members throughout the analysis process. Two lived experience researchers independently coded three transcripts and collated codes into thematic ideas. Other members of the research team (SJ, AP, NM, EP, and SR) coded one to two transcripts each, such that a total of six transcripts were coded and collectively reviewed. Additionally, another researcher independently coded and developed descriptive themes for four additional transcripts. This process was used as a validity check to compare codes and emerging themes and cross-check the themes developed by another researcher against the existing thematic framework developed by the primary researcher. All researchers coded transcripts independently except for one who had previously reviewed a draft of preliminary themes. In-depth discussions on important quotes and descriptive codes were shared to facilitate a nuanced understanding while allowing for different interpretations [37,38]. Codes arising from this initial process were developed into themes through discussions between two researchers (SI and SJ) with input from a qualitative research expert (NM). An initial thematic framework was developed by SI then discussed with the wider team for refinement through an iterative process of discussion, revision and editing. These discussions enhanced the validity of findings and promoted reflexivity by incorporating multiple perspectives at different stages of analysis. After agreement on the thematic framework, themes and subthemes were checked against primary data and descriptive codes to enhance validity of findings. Throughout this process we revised our thematic framework based on feedback, again improving validity.

## Results

Sixteen participants were recruited to this study: eight self-defining as female, five as male, two as gender fluid/non-binary and one as transgender male. Their mean age was 37 (*SD* = 12.9; age range: 20–59); and 56% of the sample self-identified as White British. Eight self-identified as heterosexual (62%). All participants reported a formal diagnosis (n = 12) of or self-identified with the diagnosis of ‘EUPD’ (n = 3), apart from one with a formal diagnosis of ‘mixed personality disorder’ (see Table 1). In the description of themes below, participants who received a formal clinical diagnosis of ‘personality disorder’ from a mental health professional are referenced after each quote as “P number” and those who self-identified with a diagnosis of ‘personality disorder’ were labelled as such “P number, self-identified with ‘EUPD’”.

**Table 1:** Participant sociodemographic and clinical characteristics (N = 16).

	Age	Ethnicity	Gender	Sexual orientation	Self-reported diagnoses
P1	50-59	White British	Female	Heterosexual	Formal clinical diagnosis of ‘EUPD’
P2	20-29	White British	Male	Bisexual	Formal clinical diagnosis of ‘EUPD’
P3	40-49	Asian/Asian British	Male	Heterosexual	Formal clinical diagnosis of ‘Mixed Personality Disorder’
P4	20-29	Asian/Asian British	Female	Heterosexual	Formal diagnosis of ‘EUPD’ Autism, Bulimia
P5	50-59	White British	Male	Heterosexual	Formal clinical diagnosis of ‘EUPD’
P6	50-59	White British	Female	Heterosexual	Formal clinical diagnosis of ‘EUPD’ Depression, seasonal affective disorder, attention deficit hyperactivity disorder, social anxiety disorder
P7	20-29	Prefer not to answer	Female	Lesbian	Formal clinical diagnosis of ‘EUPD’ Obsessive compulsive disorder, anxiety, depression, complex post-traumatic stress disorder
P8	40-49	White British	Non-binary/gender fluid	Heterosexual	Formal clinical diagnosis of ‘EUPD’
P9	50-59	White British	Male	Heterosexual	Formal clinical diagnosis of ‘EUPD’ Bipolar disorder
P10	30-39	White British	Female	Lesbian	Formal clinical diagnosis of ‘EUPD’ Anorexia nervosa
P11	20-29	Other ethnic group	Female	Bisexual	Self-identified with ‘EUPD’ Anxiety, depression
P12	20-29	Asian/Asian British	Female	Heterosexual	Formal clinical diagnosis of ‘EUPD’ Depression, anxiety, attention deficit hyperactivity disorder
P13	30-39	Black British	Transgender male	Queer	Formal clinical diagnosis of ‘EUPD’
P14	30-39	White British	Female	Heterosexual	Self-identified with ‘EUPD’
P15	30-39	White British	Non-binary/gender fluid	Queer	Formal clinical diagnosis of ‘EUPD’
P16	20-29	Asian/Asian British	Male	Heterosexual	Self-identified with ‘EUPD’

### Thematic framework

**Fig 1:**
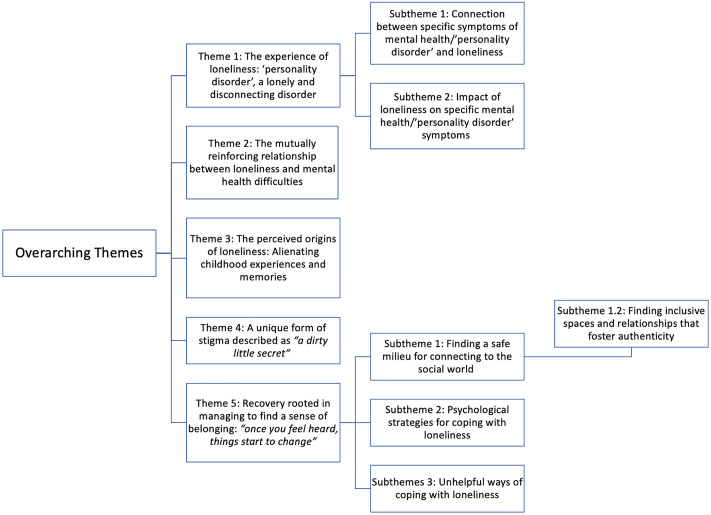
Thematic framework.

We identified five overarching themes (Fig 1). Themes 1, 2, and 3 set the context for later themes and focus on the experience of loneliness among people with a diagnosis of ‘personality disorder’, the way loneliness is perceived to be linked to mental health, and the perceived origins of loneliness as reported by participants: 1) the experience of loneliness: ‘personality disorder’, a lonely and disconnecting disorder, 2) the mutually reinforcing relationship between loneliness and mental health difficulties, and 3) the perceived origins of loneliness: alienating childhood experiences and memories. Themes 4 and 5 focus on the perceived link between stigma and discrimination and feelings of loneliness, and the ways that participants reported coping with loneliness: 4) a unique form of stigma described as a *“dirty little secret”,* and 5) recovery rooted in managing to find a sense of belonging: *“once you feel heard, things start to change”.*

S4 Appendix presents a sample of additional quotes from interview transcripts to further illustrate overarching themes and subthemes.

### Theme 1: The experience of loneliness: ‘personality disorder’, a lonely and disconnecting disorder

It was evident across all narratives that loneliness and feelings of disconnection were common, pervasive, and distressing experiences that are an inherent and integral part of who they are and their life experiences. Participants described loneliness as a sense of brokenness, a *“part of their DNA”* (P5) and felt as if they were on the “*fringes”* (P8) of society, *“less alive and vital”* (P1), misunderstood in regard to their symptoms, rejected, and unheard. Participants described a sense of disconnection, feeling that they were somehow faulty or that something was ‘*wrong with the way that my* [the] *brain functions’* (P5). P8 described this persistent feeling of loneliness, stating:

*“I guess to me it’s like a brokenness that there’s something inevitably wrong with me and that people don’t want to connect to me. But also a feeling that I can’t even if I want to”* (P8).

Underpinning several narratives was the notion that ‘personality disorder’ is in itself an enduring, lonely and disconnecting disorder. P13 described that *“BPD.. it’s a more severe condition of loneliness”* (P13).

When asked about the possible connection between loneliness and ‘personality disorder’, participants reported that loneliness is “*a major feature”* (P11) of their condition and a *“very good indicator”* and *“measure”* (P11, self-identified with ‘EUPD’) for their mental health.

A few older participants (aged 40–60) described being able to make sense of their experience of loneliness when they received a formal diagnosis and gained a better understanding of their mental health. Throughout P6’s narrative, loneliness was described as a lifelong struggle that she was finally able to understand after having received a diagnosis. She explained:

*“To be diagnosed with BPD, it’s almost like a bit like a relief because I know it’s not just me. It kind of explains to a degree why I feel lonely”* (P6).

However, two participants who self-identified with a diagnosis of ‘personality disorder’ reported that identifying with the diagnosis made “it (loneliness) worse” as they felt the need to hide their diagnosis (P11, self-identified with ‘EUPD’) or reported that they actively avoided a clinical diagnosis of ‘personality disorder’ (P14, self-identified with ‘EUPD’).

In the subthemes below, we describe and disentangle the perceived relationships between ‘personality disorder’ and mental health and loneliness, where possible. The first subtheme describes the reported ways in which loneliness is connected to, or influences, symptoms of ‘personality disorder’ and mental health. The second subtheme focuses on listing the reported ways in which mental health and ‘personality disorder’ symptoms influence feelings of loneliness.

#### Subtheme 1: Connection between specific symptoms of mental health/ ‘personality disorder’ and loneliness.

Participants expressed that specific symptoms of ‘personality disorder’, such as *“feeling empty”* (P2),*“feelings of uselessness”* (P6), a *“lack of* (a) *core identity”* (P5), *“a sense of paranoia”* (P4),*“splitting”* (P4), struggles “*with low self-esteem”* (P6) and self-worth (P1), depression, and *“dissociation”* (P14, self-identified with ‘EUPD’) influenced or/and worsened feelings of loneliness. A few participants highlighted that specific symptoms of ‘personality disorder’ such as experiencing emotions intensely and dissociative experiences made them avoid social opportunities which then contributed to ongoing and persistent feelings of loneliness. Two participants (P4; P14, self-identified with ‘EUPD’) described dissociative experiences characterised by feeling *“so disconnected from my [the] body and world”*. This led to difficulties connecting with themselves and difficulties connecting with others. P5 described his continuous struggles with a *“chronic instability in my* [his] *identity”* that contributed to dissociative experiences. He reported on the perceived direct link between these symptoms and loneliness:

*“I mean, one of the things that I kind of associated with loneliness is a sense of unreality, that my emotions are untrustworthy. And I think fundamentally what loneliness is, is it’s an emptiness and a lack of core identity”* (P5).

One participant (P16, self-identified with ‘EUPD’) explained that difficulties managing emotions such as anger had led to *“intense clashes”* that impacted his abilities to form or sustain connections. This then made him feel disappointed in himself, particularly where this conflicted with his *“perfectionist”* values, which together further intensified his feelings of loneliness.

#### Subtheme 2: Impact of loneliness on specific mental health/‘personality disorder’ symptoms.

Some participants reported that loneliness contributed to anxiety, depressive episodes, emotional dysregulation, and mood fluctuations. Participants describe intense, painful and *“deep sadness”* (P2), *“internalised anger”* (P5), anxiety and mood fluctuations resulting from loneliness and associated feelings such as feeling *“not wanted”* and burdensome*.* Despite this, a few older participants expressed that they would prefer to struggle with the depression and emotional dysregulation resulting from loneliness than experience the intense anxiety and the *“emotional chaos”* (P1) that could arise from connecting to another *“complex human”* (P1).

### Theme 2: The mutually reinforcing relationship between loneliness and mental health difficulties

In this theme, we describe the mutually reinforcing cyclical relationship between mental health difficulties and loneliness, and the perceived reasons for why and how this mutually reinforcing relationship operates. Many participants explicitly described a self-reinforcing relationship in which loneliness contributed to the worsening of mental health symptoms, which then intensified feelings of loneliness and thereon. Most participants who explicitly described this cycle between loneliness and ‘personality disorder’ symptoms were older or had engaged in self-exploration through therapy. P6, a 59-year-old female, described herself as someone who struggled with loneliness, *“relationships and being let down”* and stated that:

*“It’s like a self-fulfilling prophecy...someone lets me down, I then say ‘Right - on your bike, don’t wanna know you, see you later’. Then I feel lonely. Then I start, you know, going down with all my emotions and everything, then I isolate. And I’m back to square one where I haven’t got anybody to spend time with”* (P6).

At the core of this mutually reinforcing relationship between loneliness and ‘personality disorder’ participants often described fears of potential rejection, abandonment, social exclusion, re-traumatisation and an intolerance for unpredictability, linked to traumatic, invalidating and unsafe childhood experiences. These fears led to hypervigilance, avoidance and withdrawal thereby preventing them from attempting to achieve connections with others. P7 described how this hypervigilance contributed to feelings of loneliness:

*“I’m very perceptive, like very quickly, and sometimes make assumptions and a lot of it is like ‘does that person now hate me?’ So that can make me feel lonely very quickly. I feel like a lot of our symptoms do make us feel alone and I don’t know if that’s because we’re programmed to try and protect ourselves because we’re hypervigilant. But it almost is too much, then we feel alone. So, then we are gonna feel lonely... I think it would be a* *very core bit of BPD”.*

P4 described how such fears compromised their ability to form valued connections:

*“My expectation is that I’m going to get hurt, like, any moment. It’s a very, like, unpredictable environment to be in. So, I kind of just assume that people are gonna hurt me if I’m doing, like, anything at all slightly wrong”* (P4).

Participants who disclosed struggles with self-harm and suicidal thoughts reported that loneliness contributed either directly or indirectly to episodes of self-harm and/or suicidality through feeding into painful ideas and emotions such as shame and self-hatred. For some, these episodes of self-harm and/or suicidality would then lead to avoidance of others to hide how they feel and scars of self-harm thereby further exacerbating feelings of loneliness. P12 described this relationship as:

*“I think it was quite cyclical. Like I’d be lonely, and I’d be like basically be unable to cope with anything and then I’d like start self-harming and then I’d not want to see people. So, then it would just sort of feed into the whole cycle”* (P12).

Some participants described a cycle where missing out on socialising opportunities meant they lacked opportunities to practice interpersonal and emotional regulation skills. They felt that having engaged in these social opportunities would have helped them discover themselves and learn to emotionally self-regulate, as well as achieve a sense of belonging.

*“So, the loneliness isolated me and prevented me from learning how to manage my emotions. Then you know, as an adult... I didn’t have the tools to manage them that in turn made me avoid that* [socialising] *completely”* (P1).

### Theme 3: The perceived origins of loneliness: alienating childhood experiences and memories

Loneliness was often described by participants as originating from early childhood experiences. A range of childhood memories and experiences were referred to as the source of loneliness. These childhood experiences included difficult family dynamics, abandonment, physical, sexual and emotional abuse, illness and loss of loved ones, adoption/foster care experiences, and emotional neglect. From the range of childhood experiences described, emotional neglect, abuse or abandonment, and unsafe and invalidating childhood environments were the most commonly reported source of loneliness among participants. Some participants described these difficult experiences as having become a part of their self-identity. For example, P5 reported that loneliness is *“directly related to* [my] *childhood experiences”* and further explained:

*“I was sent to boarding school and that felt like a series of kind of rejections or abandonments.... I think that can breed a kind of a permanent state of loneliness, because you kind of feel... my parents both rejected me.... I was also sexually abused there, which I think made the whole experience even more complicated”* (P5).

Some participants shared specific experiences that exemplified their painful feelings of loneliness rooted in childhood traumatic experiences. P4 described feeling lonely her *“whole life”* and having to pretend to follow a religion for her *“own safety”* for fear of physical abuse from her family. She had felt so disconnected from her family that she had believed she must have been adopted. Eventually, she tried to take her life to *“wake up from this coma”* in the childhood belief that she would be reunited with her real loving family and finally experience a sense of belonging. As an adoptee, P9 believed he had been born a lonely infant, stating *“ I was experiencing it* (loneliness) *at not even one year old... being passed around like a parcel”* in the foster care system. P7 described feeling continuously guilty and ashamed for her childhood experiences, particularly blaming herself for a family member’s death because that person had an accident while taking care of her.

*“I was the thing that made her* (have the accident) *and I’d link the accident with the cancer and that’s what had killed her.. it’s guilt that’s kept me isolated”* (P7).

Conversely, one participant did not describe any trauma in their childhood but described herself as a *“shy”* child who experienced difficulties fitting in (P1). Other interviewees either did not mention their childhood (P8) or described childhood difficulties that they did not label as traumatic such as *“mild bullying”*, difficulties fitting in and bonding with peers, or childhood social isolation. These people tended to provide less details about their early years than other participants. Family dynamics such as having an “*absent father*” (P16, self-identified with ‘EUPD’), and cultural mindsets in which emotions were perceived as *“bad”* and must be *“swept* *under the carpet”* (P2) along with negative consequences to expressing feelings were described as invalidating childhood experiences that contributed to difficulties in identifying and expressing emotions, which then intensified feelings of loneliness.

### Theme 4: A unique form of stigma described as “a dirty little secret”

Most participants described the stigma and negative connotations of having a diagnosis of ‘personality disorder’ as key factors that played into feelings of loneliness and exclusion. Participants described being perceived as *“dangerous”*, *“monstrous”,* abnormal, *“a bad person”, “uncontrolled”,* and associated with criminality; all of which led to a sense of exclusion. Two participants described the diagnosis of ‘personality disorder’ as their *“dirty little secret”* (P8). P12 described this as a specific form of stigma that was probably more marked than that for other mental health disorders.

*“I haven’t told really any of my friends that I have this label like it’s kind of a secret. I’m way more comfortable saying I’m anxious or my mood is low, because I think there’s less stigma with that than with EUPD*” (P12).

A few participants who had shared their diagnosis with others reported feeling that those around them had distanced themselves in response. P8 described how despite putting efforts into connecting with others in the workplace, she felt that colleagues avoided her due to her diagnosis. This *“emphasised in my* [her] *mind that it was because of my* [her] *illness that they weren’t connecting with me* [her]”.

A few participants spoke about how the stigma associated with having a diagnosis/symptoms of ‘personality disorder’, coupled with other stigmatised social identities, added an additional barrier that prevented them from gaining a sense of belonging. The complex intersectionality between the stigma of ‘personality disorder’ and identifying as a sexual minority contributed to a unique experience of discrimination and exclusion that intensified loneliness. A few participants mentioned that loneliness was linked to having to hide their sexuality, religious beliefs, or values from family members and friends, with some describing having previously experienced abuse and others expressing fear of possible abuse if these aspects of their identity were revealed. Along with the stigma associated with ‘personality disorder’, a few described the sadness and anger resulting from the inability to celebrate religious festivals, Christmas, or birthdays with families and friends due to their need to maintain their secret (e.g., having a same sex partner; different beliefs/religions) and aspects of their identities that were not accepted within their families or cultures. P10 specifically described that this complex intersection between the stigma of ‘personality disorder’ and of sexuality contributed to self-hatred and shame, further intensifying symptoms of ‘personality disorder’, which they believed had led to psychiatric admission. Relating to her experiences of being raised in a strict religious group that believed that *“homosexuality was completely wrong and that anybody who had same-sex attraction needed deliverance and exorcism”,* she explained:

*“I started to kind of realise that I had feelings for women….That kind of kept cropping up, but I kept on pushing it down and that kind of made me feel more lonely and more isolated and more shame. And I think the shame increases loneliness massively….I felt a lot of shame, a lot of internalised homophobia and that led me to suicide attempts and lots of self-harm, and hospitalisations …. and just felt very lonely with that” (P10).*

P15 described having to hide their authentic self and not being able to express their sexuality. They expressed feeling like an *“imposter”* and *“rejected”*, all of which contributed to a sense of disconnection and loneliness. One participant described how having a visible physical disability was already a stigmatising and lonely experience that she could not hide. Therefore, to escape further discrimination and stigma, she had actively avoided receiving an official diagnosis of ‘personality disorder’ for fear of further mistreatment within mental health services (P14, self-identified with ‘EUPD’).

One participant who identified as a Black British Transgender Queer diagnosed with ‘EUPD’ reported intersecting experiences of discrimination and exclusion related to the diagnosis of ‘personality disorder’ along with their ethnicity during psychiatric admission that exacerbated feelings of loneliness including: being misunderstood by White clinicians and having experienced a shift in treatment after receiving a diagnosis of ‘EUPD’ which resulted in trauma associated with mental health seclusion practice and loneliness:

*“I was secluded for a period and that actually made me feel more scared about my whole self, I was being bonded* [restrained during mental health seclusion practice] *and just to prevent self-harm and harming other people …. So, I really wanted some.. display of empathy, but I think umm the people who were in charge of my entire well-being* [clinicians] *in those therapy were actually not doing what I wanted for my emotional health... I wanted to talk to people, I wanted to be normal in the community”* (P13)*.*

Overall, the majority of participants have linked their feelings of loneliness to experiences of discrimination based on their mental health and symptoms, with some expressing that this was compounded by discrimination based on other social/minority identities. Some participants from sexual minority groups described loneliness as directly connected to experiences of discrimination and feeling different from others because of their identities; with a few participants actively moving from small cities to larger cities to find an environment where they are *“accepted”.*

### Theme 5: Recovery rooted in managing to find a sense of belonging: “once you feel heard, things start to change”

Participants described the importance of gaining a sense of belonging, lifting them out of their loneliness, and how this was an integral part of their recovery. Participants expressed that loneliness had felt like being stuck in their *“own little world”* (P1), characterised by a lack of mutual and authentic connections, feeling less alive and craving connections and acceptance. Conversely, a sense of belonging was described as gaining a feeling of safety and acceptance from fulfilling and satisfying connections, a bonding experience, feeling *“genuine excitement”* (P15) and happiness, and a sense of self-acceptance. Some participants emphasised a sense of belonging as creating a sense of stability and allowing them to manage their mental health symptoms. They described moving from loneliness to a sense of belonging by learning to use psychological strategies to change their thoughts and emotions (such as self-compassion and emotional self-healing) and external approaches (such as engaging in support groups and therapy) to achieve a sense of belonging. It appeared that using these approaches could potentially break the negative cyclical relationship between loneliness and ‘personality disorder’. The narratives pointed to a virtuous cycle in which emotional processing and self-understanding fed into a sense of belonging, which improved the ability to connect with others, and thereon.

Many participants described the process of gaining a sense of belonging through the psychological strategies that allowed for self-understanding and acceptance, and a sense of connection to the self and others, and external approaches described as a journey marked by benefits and pitfalls based on trial and error and taking safe risks.

*“I think the combination of now having support systems, being able to be better at identifying my feelings and communicating them and alongside having support like my support group or like you know, my* [sibling]*”* (P10).

In the subthemes below, we describe the internal psychological and external social approaches and strategies participants reported using to alleviate loneliness as well as the perceived unhelpful ways of coping with loneliness.

#### Subtheme 1: Finding a safe milieu for connecting to the social world.

Participants described having sought and achieved a sense of belonging within a safe, validating, non-hierarchical, non-judgmental and containing social space in which they could feel comfortable and secure to explore themselves alongside others. These spaces could be therapeutic groups or peer support groups, a group activity with a meaningful purpose, an online social network, or a healing friendship.

Safety within these spaces was felt to be paramount, and acceptance of ethnic and sexual diversity, and respect for each other’s boundaries are important to nurturing a sense of safety. P11 described the importance of having experienced a sense of safety in a healing friendship that she had developed:

*“I think being safe is really important for me to feel like I belong somewhere and like I’m not alone. If I think of the people that I have now met and that make me feel like I belong, I feel safe telling them about anything about me”* (P11, self-identified with ‘EUPD’).

Similarly, P3 who is diagnosed with ‘mixed personality disorder’ described having formed a friendship that felt safe, *“almost like a security blanket”,* and made him “*not feel very lonely”*.

Volunteering and thus giving back to society was described as positive and connecting experiences. Being part of a volunteer group involving collaboration, reciprocity, and teamwork was described as a *“mutually stimulating”* (P1), and growth-promoting experiences, in which people challenged each other respectfully. Peer support work with others also diagnosed with ‘personality disorder’ was described as validating in such a context where everyone was *“just the same”* (P8), nurturing a sense of connection. Participants stated that whilst engaged in these activities they no longer felt like *“aliens”* (P8): one participant (P8) reported that *“I never feel lonely”* working as a peer support worker. Receiving peer support could also be a normalising and connecting experience: P1 described their peer support worker as someone who *“understands and they’ve gone through that same thing”*. Two participants had been involved in research, for example in public and patient involvement teams, and felt that this had reduced loneliness and promoted a sense of purpose and self-worth and described these experiences as therapeutic and rewarding.

Participants who had not found settings safe enough to establish deeper connections associated with emotional vulnerability tended to opt for casual relationships with weaker emotional ties. Restricting their relationships in this way was seen as a safeguard to protect themselves from relationship difficulties and prevent the potential for emotional pain. One participant (P16, self-identified with ‘EUPD’) described that *“one key thing I have learned is not to expect anything from anyone”*, which they described as a healthy approach to avoid disappointment.

P4 and P16 reported that they used distraction techniques to cope with loneliness, such as watching television or YouTube videos or listening to podcasts, particularly where people were interacting and discussing engaging topics, as this provided a temporary relief from loneliness.

Pets were also described as a source of comfort, safety, and warmth, which promoted a sense of connection and companionship when feeling lonely. P6, who struggled with a sense of uselessness and loneliness, described how she looked after and *“bonded”* with her pet, a living being that was comforting, *“uncomplicated”* and happy.

***Subtheme 1.2: Finding inclusive spaces and relationships that foster authenticity.*** Some participants from minority groups described finding ways to be their authentic selves and attaining a sense of belonging through living a life of authenticity and *connecting “to others as my [their] true self”* (P11, self-identified with ‘EUPD’). This was achieved through finding *“an environment that is accepting of my* [their] *sexuality”* (P10) or deciding to share their sexual preferences with family members regardless of potential ramifications or with specific family members who are more accepting. One participant described that moving to a new city with a large Queer community made her feel *“accepted and even celebrated”* and *“created a huge sense of belonging”* (P11, self-identified with ‘EUPD’). P15, who identified as Queer and neurodivergent, noticed that they felt a strong sense of connection to themself and others when around their *“found family”,* a group of people who were also Queer and neurodivergent.

However, this was found to be met with challenges. For instance, P15 described that despite feeling/perceiving themselves as accepted and having genuine connections, their ingrained responses to interpersonal relationships included *“cruel”* and unhelpful core beliefs and *“intrusive thoughts”* about being undeserving of friendships shaped by past trauma:

*“I just have people in my life who are genuinely excited to see me and to be around me. It’s just I think it’s because I’m doing good things for myself and it’s scary and it’s stabilising. My brain is reacting with old messages that are not helpful that are not true in the space and time, feeling more lonely”*

#### Subtheme 2: Psychological strategies for coping with loneliness.

Some people described coping with loneliness through their own forms of self-help such as physical activities and reconnecting with nature. These alleviated loneliness by promoting a sense of belonging (where this involved being alongside others), accomplishment and confidence, but also through creating a connection to oneself by helping with emotional regulation. P12 described this sense of accomplishment:

*“I climbed* [a mountain] *a few years ago and I think that was like the least lonely because you’re surrounded with people and you are all doing something very challenging and like pushing your body”*.

In some cases, this prevented incidents of self-harm. For instance, P11 (self-identified with ‘EUPD’) described that when overwhelmed with feelings of loneliness, she engaged in art and photography as opposed to self-harm. P12 described hiking and running as “*a tool to stop immediate self-harm”* and “*to feel like good about yourself”.*

Where participants reported achieving a sense of belonging and connection, this tended to relate to engagement in creative interests such as arts, music and choir, and sports. Sometimes, these could be activities that took place alongside others, even where all were focused on the task at hand rather than a group activity. For instance, P2 reported that when he was admitted to a psychiatric ward, he had *“found it really helpful to sort of draw out how you feel”* alongside others with similar mental health issues and discovered that it was a *“very good way of coping”.* Participants described that these activities promoted a sense of purpose and a “*sense of flow”* and were meaningful.

*“Things that sort of helped with my loneliness was playing sports with other people, so tennis, and whether it’s just because of the sense of flow, the sense of competitiveness of fun and, you know, a bit of banter.... I think being in a state of flow is really good at pushing loneliness out of the picture”* (P5).

Some participants identified personal interests such as writing, journalling, and drawing in the course of therapy or discovered them on their own, reporting that they provided a helpful way of expressing emotions and connecting with themselves. A few participants described using meditation and yoga and mentally immersing and engrossing themselves in activities. For instance, P9 stated:

*“I would engross myself into that place where* [a specific autobiography was set], *and by doing that, I think I need to be in that safe place in order to look at and to reassure myself off my loneliness”.*

Participants also mentioned using grounding techniques, using self-soothing boxes consisting of their favorite scents/pictures, positive self-talk, self-compassion and self-care to cope with loneliness when it became intense. These skills were often described by participants who had engaged in group therapy such as dialectical behavioural therapy (DBT) or individual therapy.

*“So for example, if I’m feeling abandoned or lonely, then I kind of...I go to myself and like, OK, what am I actually feeling and then like try to umm sort of self-soothe myself”* (P2).

Other participants learned to identify and step away from situations that might prompt feelings of loneliness and had conversations with themselves to understand (and potentially communicate to others) their feelings. They felt that this deeper sense of self-awareness and reflection on the root causes of their loneliness contributed to better relationships with themselves and others. This level of self-understanding and compassion was often achieved through individual therapy work. P10 described learning to be kinder to herself by *“reparenting*” herself, acknowledging and processing painful emotions, and expressing them which allowed her to connect with others as her true self. She learned how to identify her different feelings and express them to others. Participants who had not had opportunities to engage in these therapies aspired to gain access and believed it would help them better cope with loneliness and their mental health symptoms.

#### Subtheme 3: Unhelpful ways of coping with loneliness.

Participants identified ways of coping with loneliness that they acknowledged were either ineffective or only temporarily effective. Two participants described a tendency to self-isolate or bury themselves in work to avoid opportunities to form connections and suppress their need to belong. However, they were also aware that these were ineffective and counterproductive.

Some participants described attempting to numb and control the pain of loneliness by using alcohol, engaging in disordered eating patterns, obsessively working out, self-harming or befriending people who reminded them of their abuser.

*“I think that sense of loneliness, for me, the easiest thing I could do was drink to just…It’s basically it was to numb the loneliness. That’s exactly why I drank as much as I did, I suspect.”* (P5).

*“I guess like if I’m feeling more lonely, then I go to my eating disorder to get this sort of sense of control”* (P2).

P15 described hiding their authentic self and noticing a tendency to befriend people who had similar characteristics to that of their abuser, seeing this as a way of understanding the abuser’s motivations, hoping that this would help them make sense of their past abusive experiences:

*“but there was a lot of unsafe behaviour there. So, it became sort of a pattern of me trying to reach out to people like my dad to try and work out what that connection meant”.*

Participants observed these strategies as predominantly unsuccessful (and counterproductive) in helping them gain a sense of belonging. The distinction between helpful and unhelpful ways of coping with loneliness was not always clear: one participant mentioned that work can sometimes negatively impact their sense of belonging as well as functioning as a good distraction from loneliness (P12). While it is not clear whether there are age-related patterns, individuals described having been used and abandoned these unhelpful ways of coping when younger, and some people younger in age reported currently using these methods (e.g., self-harm).

## Discussion

### Main findings

The findings from this qualitative study exploring the experiences of loneliness among people with a diagnosis of ‘personality disorder’ identified five overarching themes, all demonstrating the experience and impacts of loneliness and ways of coping with it.

Our findings indicate that chronic loneliness is experienced as a pertinent, painful and enduring issue for many people with a diagnosis of ‘personality disorder’, with participants suggesting that loneliness is an indicator of the severity of their symptoms and a core feature of ‘personality disorder’ (Theme 1). Participants also described what appeared to be a mutually reinforcing relationship between loneliness and ‘personality disorder’ features in which loneliness worsened mental health symptoms, and vice-versa (Theme 2). Many people perceive their loneliness as a painful experience that is often rooted in alienating, invalidating - and at times traumatic - childhood experiences and memories that continue to linger into adult years (Theme 3). The stigma associated with the diagnosis was a key barrier to gaining the sense of belonging that people with a diagnosis of ‘personality disorder’ yearned for, sometimes compounded with discrimination based on minority identities and protected characteristics (Theme 4).

Participants described a range of strategies employed to cope with and manage feelings of loneliness (Theme 5). These were described as a combination of psychological therapeutic work, learnt coping skills (e.g., DBT skills) and an emotional journey towards self-understanding and healing (e.g., emotional exploration), and finding safe social spaces and activities. Some participants used what they saw as unhelpful and temporary ways to cope with loneliness (e.g., self-harm/alcohol use), or perceived drawbacks in some approaches that they also found helpful (e.g., burying themselves in work).

It is important to note that these findings are primarily based on the experiences of loneliness among people with a diagnosis of ‘EUPD’.

### Findings in the context of other studies

Participants in our study described that loneliness was perceived to originate from early childhood experiences that had been invalidating and/or traumatic. These experiences continued to shape ongoing feelings of disconnection and loneliness. These findings supported and expanded upon the preliminary findings from our previous meta-synthesis on loneliness among people with a diagnosis/traits of ‘personality disorder’, which highlighted that a sense of disconnection seemingly arises from childhood experiences of invalidation and interpersonal traumatic events [1]. These findings could be explained in the context of attachment theory and the biosocial theory of emotional regulation, both of which are commonly used in understanding the development of ‘personality disorders’. Attachment theory suggests that relational issues and associated issues (e.g., fear of others) stem from underlying insecure attachment patterns resulting from early invalidating and traumatic childhood experiences [39]. Many studies found that people with a ‘personality disorder’ diagnosis develop insecure attachment styles which are linked to ongoing fear of abandonment, intimacy and rejection, struggles with expressing and regulating emotions, and avoidance of emotional closeness to others [39]. The biosocial theory further postulates that these early invalidating experiences intersect with predisposing biological risk factors for emotional sensitivity contributing to difficulties understanding and regulating emotions, increase emotional sensitivity and impulsivity, and negative beliefs about the self and others; all of which impact ability to form and maintain healthy relationships [40].

As demonstrated by our findings, ‘personality disorder’ and loneliness may share similarities that play into a self-perpetuating cyclical relationship where loneliness and clinical features of ‘personality disorder’ mutually reinforce each other. This is corroborated by findings from a review on the association between loneliness and ‘personality disorder’ traits that suggested that ‘personality disorder’ and loneliness share overlapping intrapersonal (e.g., emotional dysregulation, fear of rejection) and interpersonal (e.g., social avoidance) factors [3]. These shared features suggest that loneliness may be a central feature of ‘personality disorder’ that appears to contribute to the worsening and persistence of symptoms.

Our findings demonstrated that the stigma of and negative connotations associated with ‘personality disorder’ created a lonely and exclusionary existence that cultivates a sense of disconnection from society. There is evidence that people with a diagnosis of ‘personality disorder’ face more stigma than other psychiatric diagnoses such as depression [7]. It appears that loneliness manifests differently among people with a diagnosis of ‘personality disorder’ because it interacts with a unique form of stigma associated with ‘personality disorder’ and, for some, this is compounded by discriminatory experiences based on other protected characteristics.

Loneliness, coupled with the wider societal barriers and experiences of discrimination, further intensifies feelings of loneliness and adds another burden for people with ‘personality disorder’ diagnoses. This is further complicated by the stigma and negative social perceptions associated with protected characteristics and intersectional social identities that, combined with the stigma of ‘personality disorder’, add another layer to loneliness, intensifying it further. Participants described discrimination and stigma associated with protected characteristics (e.g., sexuality) in relation to emotions of shame, inadequacy and guilt, all of which were linked to loneliness. Previous quantitative cross-sectional and prospective studies exploring stigma and mental health outcomes among people from minority groups found that people from minority groups experience high levels of self-hatred and shame which is linked to shame-related psychological difficulties (e.g., depression/suicidal thoughts) and coping behaviours (e.g., concealment/withdrawal) [41–43]. The intersectionality between minority status(es) and stigma associated with one’s mental health status, particularly for the stigmatising diagnosis of ‘personality disorder’, is seen to contribute to intense shame and consequent attempts to conceal the identity; this poses a barrier to forming connections and increases loneliness [44].

Our finding that some participants in our study used self-harm as a means of (temporarily) coping with loneliness in the context of ‘personality disorder’ is consistent with other qualitative research with adults diagnosed with ‘EUPD’ which identified self-harm as one strategy used to manage loneliness, experienced as painful feelings of emptiness, even if this strategy carried risks [18]. We noted that many of the strategies found helpful in this study to address loneliness, such as self-soothing/grounding skills, distraction and challenging negative thoughts, were similar to those involved in Dialectical Behaviour Therapy (DBT); an established third wave psychotherapy for addressing difficulties experienced in ‘EUPD’ [45]. Although DBT does not explicitly target loneliness, it can be seen why DBT is helpful as a foundation for people with ‘EUPD’ when experiencing intense periods of loneliness [45]. Participants also described using distraction techniques and alternatives to self-harm to help them cope with overwhelming feelings of loneliness, as a way to reduce their self-harm. Research on appropriate responses to self-harm suggests that a personalised approach, in which the function of self-harm is considered and explored (such as identifying that self-harm can arise from loneliness) can inform the type of support offered to people who self-harm [46,47]. Research exploring the reasons and motivations behind self-harm demonstrates that people use self-harm as a way to manage distress, destructive thoughts and dissociation, all of which are relevant issues in ‘personality disorder’ [46–48]. Many of these difficulties were described by participants in our study. Substitutes for self-harm identified by participants in this study were meditation, physical activities, creative activities, self-care, connecting with supportive people whom they can be authentic with, and helping others. This is consistent with the distraction techniques and alternatives to self-harm identified in the literature on self-management of self-harm [47,48].

Participants described that managing feelings of loneliness and building a sense of belonging was an integral part of their recovery. The literature on ‘personality disorder’ and recovery supports the notion that recovery is largely relational [1,14,49]. We further expand upon these findings in the implications section below.

### Strengths and limitations

This qualitative study expanded upon a topic that has been overlooked in the literature on ‘personality disorder’. To our knowledge, this is the first qualitative study that recruited a diverse sample of people across multiple identity groups, including ethnicity, gender, and sexuality. Lived experience was incorporated at each stage of the study, including refining the research questions, designing the topic guide and recruitment plan, analysis of a proportion of data, and discussing the implications of findings. The involvement of a multidisciplinary team of researchers and clinicians in independently coding transcripts and in theme development and refinement further enhances the validity of findings. Furthermore, participants were at various stages of recovery, with some participants having not received any treatment or awaiting treatment and others in treatment. Recruiting people at various stages of recovery was a strength in that it portrayed a variation of experiences and the impacts of treatment on experiences of loneliness.

Although a multidisciplinary team were involved throughout the study, interviews with all participants and analysis were primarily carried out by one researcher, which could shape how the interviews were conducted, potentially influencing the participants’ responses due to social desirability or other biases. Although this approach can impact the validity and credibility of our findings, we have taken steps and put measures in place to reduce the risks of these biases (see S5 Appendix). Another limitation was that we recruited primarily interviewees with a diagnosis of ‘EUPD’ and our findings therefore relate primarily to people with this label and should not be generalised to those with other types of ‘personality disorders’. There are different philosophical frameworks that could have been used in this study and in particular one approach that could have been adopted in this study is that of social constructivism, which stipulates that knowledge is a result of a process of *learning* that takes place in interactions and that there are diverse interpretations that could be learned [50]. This approach could have offered different insights, although we had rejected these because of criticisms that they may be too restrictive “by only seeing the truth as a social convention, playing by the rules of a particular group” [51].

### Clinical and research implications

The findings from this study support the need to co-develop and produce – in collaboration with people with relevant lived experience - a set of psychosocial interventions that target loneliness among people with a diagnosis of ‘personality disorder’.

Participants described a range of group and individual one-to-one activities that helped alleviate loneliness, including peer support work (receiving/giving), involvement in support groups, volunteering, and shared interest-based activities. However, such group activities must be characterised by shared group values and be perceived as safe and accepting to allow for emotional exploration and authenticity. Quantitative cross-sectional and prospective studies have found that social identification through group membership promotes a sense of purpose, self-esteem, and connection [52–54]. Developing a self-identity often involves understanding one’s place in the world - how one relates to others, to social groups, and to broader societal structures [52,53]. Our findings suggest that a socially focused intervention could potentially reduce loneliness in this clinical group through developing a sense of social identity and group belonging [55,56]. Our qualitative findings highlight the importance of a person-centred and flexible approach tailored to the specific individual’s needs, preferences, and assets (e.g., nature, physical activity). Furthermore, future research should explore experiences of loneliness among people with different types of ‘personality disorder’ diagnoses as different ‘personality disorders’ may have differing reasons and factors associated with loneliness. For instance, people with a diagnosis of ‘avoidant personality disorder’ may be lonely due to avoidance and fears of rejection while people with a diagnosis of ‘EUPD’ may feel lonely due to finding interpersonal relationships distressing and feelings of emptiness [3].

Incorporating a psychologically focused component addressing the psychological processes and barriers maintaining loneliness can potentially strengthen self-confidence, enhance engagement in social opportunities, and improve ability to cope with intense emotions associated with loneliness [57,58]. This is particularly important when working with people with intersecting minority identities and who face discriminatory experiences. Participants from minority groups had found that living a life of authenticity and presenting one’s true self was the foundation for gaining emotional closeness and belonging. It would be beneficial for future research to investigate the mechanisms of change among people with a diagnosis of ‘personality disorder’ who had engaged in some form of psychosocial intervention (e.g., peer support/group therapy) intended to directly or indirectly alleviate loneliness. Furthermore, future research should consider involving lived experience experts in all aspects of their studies including data collection and conducting interviews, and potentially incorporate member checking to enhance the credibility and internal validity of findings [59].

Pending future research and development of a set of interventions targeting loneliness for this group, it is important for services to routinely ask service users about feelings of loneliness and explore ways of managing loneliness. This is particularly important given the growing calls to broaden intervention to include addressing social and relational needs.

### Conclusion

This qualitative study explored the experience of loneliness and coping skills used to manage loneliness among 16 people with a diagnosis of ‘personality disorder’ in the UK from different ethnicities, genders, sexualities, and backgrounds. Our findings suggest that persistent and enduring feelings of loneliness are perceived by participants to originate from difficult, traumatic, and invalidating childhood experiences. These persisted into adulthood and contributed to experiences of disconnection and loneliness, and worsening of mental health symptoms. A mutually reinforcing relationship between clinical features of ‘personality disorder’ and loneliness is evident in which loneliness exacerbates ‘personality disorder’ features, and *vice-versa*. Discriminatory experiences based on minority identities and the stigma associated with ‘personality disorder’ led to a sense of exclusion and served as an additional barrier to achieving a sense of belonging. A combination of valued group-based activities and safe and healing social relationships alongside learned psychological skills and emotional exploration was used to manage feelings of loneliness. Given the centrality of loneliness in this clinical group, it is important to co-develop and produce effective interventions tailored to the social needs and social difficulties faced by people with a diagnosis of ‘personality disorder’.

## Supporting information

S1 Appendix Co-designed topic guide. (DOCX)

S2 Appendix Qualtrics questionnaire form. (DOCX)

S3 Appendix Consent form and information leaflet. (DOCX)

S4 Appendix Themes, subthemes, and illustrative quotes. (DOCX)

S5 Appendix Reflexivity and areas of discussion. (DOCX)
