# Supplementary material for: Experiences of loneliness among people with a diagnosis of ‘personality disorder’: A qualitative interview study in the UK
Source: PLOS Ment Health. 2026 Aug 10;3(8):e0000671. doi: 10.1371/journal.pmen.0000671 (PMC13456265; doi:10.1371/journal.pmen.0000671)
Supplement: Supplementary file 1 [file pmen.0000671.s001.docx]

# **Appendix 1: Co-designed topic guide for interview**

**Part 1: What is loneliness:**

- Can you tell me what the word lonely means to you?
  - Prompts: How would you define loneliness? How does loneliness make you feel physically, emotionally, and psychologically?
- Is loneliness a significant issue for you? Could you tell me a bit more about that?

**Part 2: Experiences of loneliness:**

- You mentioned when I screened you for this study that you identified with having had experiences of feeling lonely. Why do you think you get lonely?
- Are there things that you think are linked to your feelings of loneliness?
  - Prompts: (prefaced by ‘this may or may not be the case for you’) age, culture, sexuality, personality characteristics, difficulty fitting in, stopping work, moving location, loss of a partner or family.
- This may or may not be the case for you, sometimes people identify specific or general events that may be linked to current feelings of loneliness. Can you think back to an early memory when you realised you might be feeling or experiencing loneliness?
- How do these feelings of loneliness connect to your day-to-day life?
- Are there any situations during which feelings of loneliness are particularly prominent?
  - Prompts: Times (seasons)? Events? Places?

**Part 3: Loneliness and ‘personality disorder’ or complex emotional needs:**

- Do you think feeling lonely is connected to your mental health/symptoms of complex emotional needs (‘personality disorder’)?
  - If yes, in what ways?
- Do any symptoms associated with complex emotional needs (‘personality disorder’) link to feelings of loneliness?
- Thinking back to when your mental health problems first started, do you have any thoughts on whether feeling lonely came before or after or whether they developed together?
  - [If loneliness came first]: Has your experience of loneliness changed since having mental health problems?

**Part 4: Coping with loneliness in the context of complex emotional needs or ‘personality disorder’:**

- From your lived experience of complex emotional needs (‘personality disorder’), what skills/approaches do you use when you feel most lonely in order to feel less lonely?
  - Prompts:
    - Do you have any coping techniques you use?
    - Do you have any relationships or friendships that you feel reduce loneliness? What is it about these relationships/friendships that makes you feel less lonely?
    - Have you had support with this? What kind of support? Has this helped?
    - Have you had any community or neighbourhood resources that helped you feel less lonely?
    - Can you describe an occasion in which you felt less lonely?
- What do you think others with complex emotional needs (‘personality disorder’) can do to reduce their feelings of loneliness?
  - Prompts: Psychological or social skills?
  - What advice would you give a friend?
