# Supplementary material for: Experiences of loneliness among people with a diagnosis of ‘personality disorder’: A qualitative interview study in the UK
Source: PLOS Ment Health. 2026 Aug 10;3(8):e0000671. doi: 10.1371/journal.pmen.0000671 (PMC13456265; doi:10.1371/journal.pmen.0000671)
Supplement: Supplementary file 2 [file pmen.0000671.s002.docx]

# **Appendix 2: Qualtrics questionnaire form**

**What is your full name?**

**Could you provide your phone number for contact**

**What is your age?**

**Please specify your Ethnicity**

English, Welsh, Scottish, North Irish, British

White Gypsy or Irish Traveller

Roma

Other White

Irish

Asian/Asian British

Pakistani

Indian

Bangladeshi

Chinese

Other Asian Background

Black/Black British

Caribbean

African

Any other Black, Black British, or Caribbean background

Arab

Mixed: White and Black Caribbean

Mixed: White and Black African

Mixed: White and Asian

Any other Mixed or multiple ethnic background

Other Ethnic Group

Prefer not to answer

**Could you provide your email address**

**How would you describe your gender?**

Male

Female

Transgender male

Transgender female

Non-binary/Gender fluid

Prefer not to answer

Not listed

**Which of the following do you identify with?**

Bisexual

Heterosexual/straight

Gay/lesbian

Asexual

Queer

Pansexual

Other

Prefer not to say

**How would you best describe the area you live in?**

Inner City Area

Suburban Area

Town

Village

Rural/countryside

Prefer not to say

**Please pick one of the following statements**

I self-identify with the symptoms/diagnosis of complex emotional needs or 'personality disorder'

I have been diagnosed with complex emotional needs or a 'personality disorder'

**What 'personality disorder' do you identify as having or have been diagnosed with?**

**Are you diagnosed with any other mental health conditions? If yes, please specify.**

Yes

No

Prefer not to answer
