# Supplementary material for: Experiences of loneliness among people with a diagnosis of ‘personality disorder’: A qualitative interview study in the UK
Source: PLOS Ment Health. 2026 Aug 10;3(8):e0000671. doi: 10.1371/journal.pmen.0000671 (PMC13456265; doi:10.1371/journal.pmen.0000671)
Supplement: Supplementary file 3 [file pmen.0000671.s003.docx]

# **Appendix 3: Consent form and information leaflet**

**Participant Information Leaflet/Sheet**

**Division of Psychiatry**

**Faculty of Brain Sciences**

UCL Research Ethics Committee Approval ID Number: 23645/001

Title of Study:

Exploring Loneliness among people with ‘Complex Emotional Needs’ (CEN): A Qualitative Study

**Department:** Division of Psychiatry, Faculty of Brain Sciences

**Name and Contact Details of the Researcher(s):** Sarah Ikhtabi, [sarah.ikhtabi.19@ucl.ac.uk](mailto:sarah.ikhtabi.19@ucl.ac.uk) Alexandra Pitman, [a.pitman@ucl.ac.uk](mailto:a.pitman@ucl.ac.uk)

Nicola Morant, [n.morant@ucl.ac.uk](mailto:n.morant@ucl.ac.uk)

Name and Contact Details of the Principal Researcher:

Sonia Johnson, [s.johnson@ucl.ac.uk](mailto:s.johnson@ucl.ac.uk)

1. Invitation Paragraph

You are being invited to take part in a qualitative interview study exploring the experience of loneliness among people with ‘complex emotional needs’, who may be also diagnosed with a ‘personality disorder’. Before you decide on whether you would like to be involved in this interview study, it is important for you to understand why the research is being done and what participation will involve for you. Please take your time to read the following information carefully and discuss it with others if you wish. Ask us if there is anything that is not clear or if you would like more information. Take time to decide whether or not you wish to take part.

Thank you for reading this.

The findings of this project will be used by the PhD research student, Sarah Ikhtabi, to obtain a PhD in Psychiatry in the Division of Psychiatry at University College London.

2. What is the project’s purpose?

Loneliness is a painful emotional state characterised by a gap between the quality and quantity of the social relationship we **have** and those that we **desire to have**. Research shows that loneliness is a common and difficult experience among people struggling with mental health problems, particularly among people with ‘complex emotional needs’. This is problematic as positive relationships are essential in the recovery of people with ‘complex emotional needs’, yet we lack research on how to help people with ‘complex emotional needs’ deal/cope with loneliness in their lives. Therefore, it would be essential for studies to explore loneliness as a possible treatment target.

The first step towards developing an intervention to support people with ‘complex emotional needs’ experiencing loneliness would be to gain a better understanding of loneliness and to explore loneliness-related experiences. In this one-on-one interview, we aim to explore your experiences of loneliness, including your perceptions of the origin of your feelings of loneliness, and what factors reduce or worsen feelings of loneliness in your life. The information identified through this research will inform future ways of supporting people with ‘complex emotional needs’ in reducing feelings of loneliness and improving their wellbeing.

This study will take place from September 2022 to September 2023.

3. Why have I been chosen?

In this study we are interviewing people aged 18 or older who self-identify as having ‘complex emotional needs’ or a ‘personality disorder’. This will help provide an understanding of loneliness in the lives of people with “complex emotional needs”.

To be able to take part in this study you must: 1) self-identify as having either traits or a diagnosis of ‘complex emotional needs’ or ‘personality disorder’, 2) have experienced feelings of social disconnection and loneliness, 3) be above 18 years of age, 4) be located within the UK and 5) speak fluent English.

We are aiming to recruit 15 participants, representing a range of ethnic, gender and sexual identities.

4. Do I have to take part?

Your participation in this research project is entirely up to you. ***‘It is up to you to decide whether or not to take part.*** If you decide to take part and later change your mind, you are free to withdraw from the project at any stage during the interview without any loss of benefits. If you do decide to take part, you will be given this information leaflet to keep and you will be asked to sign an online version of a consent form. You can withdraw at any time without giving a reason or explanation. If you decide to withdraw your consent during the interview or up to one month ***after the interview is conducted***, then all your data you have provided up to that point will be deleted immediately.

Please note that if you do choose to participate in this interview study, the interview will be audio recorded.

5. What will happen to me if I take part?

If you express interest in this study, you will be invited to participate in a one-on-one interview led by the researcher, Sarah Ikhtabi, at your chosen convenient date and time. We will then book an interview time and date together. The interview is a single interview and will take place via Microsoft Teams.

The interviewer will send you the Microsoft Teams link or a phone number linked to Microsoft Teams, to call in with, at least 24 hours before the set interview date and time. The interview will last approximately 1 hour and 20 minutes. The consent form will be sent to you before the interview to read through on your own time and pace. You will also be sent a form to complete and send back prior to the interview.

During the first 15 minutes of the interview, we will talk through this information leaflet and the consent form together and any potential questions or concerns you may have will be discussed and answered. If you are happy to proceed, then the researcher will read out the statements in the consent form and we will ask you to indicate whether you agree to each one. This verbal consent will be audio recorded.

The interview will take place after this and will last up to 1 hour. No one else but the interviewer will be present during the interview unless you would like someone else to be there with you. You are free to refuse to answer any question you wish to skip, without providing any form of explanation. At any point during the interview, you can ask the interviewer to pause or stop the interview completely if you wish to do so and the interviewer will move on to the next question. We can take as many breaks as you would like throughout the interview. Throughout, the researcher will ask you questions about your experiences with loneliness and related experiences, including the origin of your loneliness, times when loneliness is particularly prominent for you, and times when loneliness is less pronounced, and the ways you cope with loneliness. You can share as much or as little as you would like for each of the questions asked. The interview will be audio recorded, as this is a requirement for this study, then typed out/ transcribed in full by the researcher who conducted the interview. The data will be made completely anonymous, which means that all potentially identifying information will be removed, and it will not be possible for anyone to link it back to you. The researcher will start transcribing the interview recording a week after the interview takes place.

6. Will I be recorded and how will the recorded media be used?

The Microsoft Teams interview will be audio recorded. It is a requirement for the interview to be audio recorded for this study. If you choose to keep your camera on during the interview, then the video will not be recorded. This audio recording will be kept confidential and no one, other than the researcher of this study and the principal researcher involved in this study, will have access to the audio recording. The recording will be stored on a secure, password-protected university folder and destroyed immediately after the researcher finishes her PhD, on October 1, 2025. The audio recording will also be transcribed (typed out in full) and any personal information or identifiers will be excluded from the transcription. The anonymized and de- personalized transcription will be analysed, and the researcher may choose to publish some direct quotes to illustrate key themes but these will not identify anyone. The de-personalised transcripts will also be stored in a password protected and secure UCL online system. The transcribed and anonymized data will be only accessible to members of the project team.

No one other than the researcher conducting the interview and the principal researcher will have access to the audio recordings.

Personal information and special category data provided, including name, phone number, email address, age and gender, sexual orientation, ethnicity, whether diagnosed or self-identifying with ‘complex emotional needs’ or ‘personality disorder’, and the type of ‘personality disorder’, will also be deleted after the researcher finishes her PhD, on October 1, 2025. Alongside the audio recording, this personal information and special category data will be stored on a secure, password-protected university folder.

7. What are the possible disadvantages and risks of taking part?

The interview will take up to 1 hour and 20 minutes of your time. During this time, you will be asked to share some personal information as described above. This may be uncomfortable or potentially upsetting to talk about and may potentially remind you of an unpleasant experience. You do not have to answer the questions and can share only as much as you are comfortable sharing.

At the end of the interview, the researcher will ask you about your experience and provide you with a list of confidential phonelines and support resources that are accessible to you such as Samaritans and MIND. Should you feel like you may require extra support, you can ask for this at the time, or contact the principal researcher, Sonia Johnson, or fellow researcher Alexandra Pitman. You will also be offered the option of a follow up phone call or email to check on how you are doing within the next few days following the interview.

8. What are the possible benefits of taking part?

While there will be no immediate and direct benefits from participating in this project, your participation would help us learn more about the impact and role of loneliness among people with ‘complex emotional needs’. This would advance our knowledge on loneliness and inform the future development of interventions that would target loneliness-related experiences and issues associated with or resulting from loneliness. It is hoped that a better understanding of loneliness in the lives of people with ‘complex emotional needs’ would also guide the focus of future research and guide clinicians, therefore improving the recovery outcomes. If you would like, the researcher can also send you a transcribed and anonymized copy of the interview transcript for your own reflection.

9. What if something goes wrong?

If you have any complaints or concerns about any aspect of this study, please contact the principal researcher, Sonia Johnson (email: [s.johnson@ucl.ac.uk](mailto:s.johnson@ucl.ac.uk)) to raise your concern. If you have any specific inquiries regarding the study itself, please feel free to contact Sarah Ikhtabi, [sarah.ikhtabi.19@ucl.ac.uk.](mailto:sarah.ikhtabi.19@ucl.ac.uk) If you do not feel satisfied with the handling of any of your concerns, you may also contact the Chair of the UCL Research Ethics Committee via email – [ethics@ucl.ac.uk.](mailto:ethics@ucl.ac.uk)

10. Will my taking part in this project be kept confidential?

All the information that we collect about you during the research will be kept strictly confidential. Audio recording of your consent to take part in this study and your audio recorded interview will be kept in a separate and password protected folder stored in the protected UCL and General Data Protection regulation (GDPR) approved online system. Transcripts will be anonymized so that you would not be recognized from any information you have provided during the interview. To do so, the researcher will exclude any personal identifiers when transcribing the interview. The final transcribed version stored will not include any personal information and your name and any other personal data will be replaced when recording is transcribed. You will not be able to be identified in any ensuing reports or publications. The audio recording of the interview will be immediately deleted after the researcher finishes her PhD on October 1, 2025. The interview transcription, verbal audio consent recording and written consent form will be kept for 10 years, in line with UCL guidance, and then be deleted afterwards. Only the researchers of this study can have access to these recordings and transcriptions. These transcripts, verbal audio consent recording and written consent form will be kept safely in a password-protected university folder. The anonymized transcribed scripts will only be accessed by the researcher’s supervisor and research team, as necessary.

If researchers would like to use a direct quote from you in a publication, they will use a fake name or random participant number. Confidentiality will be respected, **however if there are compelling reasons for the interviewer to believe that you or someone you know is at direct risk of harm, confidentiality could be breached, and relevant agencies will be contacted (see below).**

11. Limits to confidentiality

Please note that confidentiality may not be guaranteed, due to the limited size of the participant sample.

Please note that confidentiality will be respected as far as possible, unless there are compelling and legitimate reasons for this to be breached. This includes the following:

If during our conversation the researcher hears anything which makes them worried that someone else or yourself might be in danger of harm. In this case, the researcher might have to inform relevant agencies of this. You will be informed of this prior to the researcher informing relevant agencies, if this is necessary.

Evidence of potential harm or crime has been uncovered.

It is important to note that there are also limits to confidentiality associated with using Microsoft Teams. All personal information required for the study will be collected using a form, before the meeting takes place on Microsoft Teams.

12. What will happen to the results of the research project?

The data collected in this study will be analysed using the approach of thematic analysis. The study results will be published in a relevant journal and presented within the PhD researcher’s thesis. The findings will also be presented at relevant research meetings. You will not be identified in any publication however we may publish direct quotations that are anonymized. The data gathered in this study will be stored securely on password protected folders within UCL’s secure online system. This paper will also be summarised in a short blog and published on Mental Elf, a website that promotes accessibility of research material. You may also be sent a summary report of the study findings prior to publication- please let the researcher know if this were something you would like. You will also be given the option to retain your contact details so that you would be informed of future participation opportunities in research projects in the future. This is optional and if you do not wish to be contacted regarding future studies, please select ‘no’ for this section on the consent form.

13. Local Data Protection Privacy Notice

The data controller for this project will be University College London (UCL). The UCL Data Protection Officer provides oversight of UCL activities involving the processing of personal data, and can be contacted at [data-protection@ucl.ac.uk](mailto:data-protection@ucl.ac.uk)

This ‘local’ privacy notice sets out the information that applies to this particular study. Further information on how UCL uses participant information can be found in our ‘general’ privacy notice by clicking here.

The information that is required to be provided to participants under data protection legislation (GDPR and DPA 2018) is provided across both the ‘local’ and ‘general’ privacy notices.

The categories of personal data used will be as follows:

Name

Phone number

Age

Email Address

Gender

Ethnicity

Sexual Orientation

Whether you are diagnosed or self-identify with “complex emotional needs” or a “personality disorder”

What “personality disorder” do you identify as having or have been diagnosed with?

Collecting these personal data will help us in gaining a comprehensive understanding of the different perspectives given by participants. The lawful basis that will be used to process your personal data are: ‘Public task’ for personal data and’ Research purposes’ for special category data.

*Your personal data will be processed so long as it is required for the research project*. The personal data you provide in this study will be anonymised and we will minimise the processing of personal data wherever possible. If you are concerned about how your personal data is being processed, or if you would like to contact us about your rights, please contact UCL in the first instance at [data-protection@ucl.ac.uk.](mailto:data-protection@ucl.ac.uk)

14. Who is organising and funding the research?

This study is organised within University College London, Division of Psychiatry.

The Economic and Social Research Council (ESRC) are funding the PhD student undertaking this research study.

15. Contact for further information

If you have any questions and/or concerns, please contact Sonia Johnson (email: [s.johnson@ucl.ac.uk](mailto:s.johnson@ucl.ac.uk)) or Alexandra Pitman ([a.pitman@ucl.ac.uk](mailto:a.pitman@ucl.ac.uk)) or Sarah Ikhtabi ([sarah.ikhtabi.19@ucl.ac.uk](mailto:sarah.ikhtabi.19@ucl.ac.uk)).

Division of Psychiatry, Maple House, 149 Tottenham Court Rd, London W1T 7NF.

You will be provided a copy of this information leaflet and consent form to keep. Thank you for reading this information leaflet and for considering taking part in this research study. All participants will be given a £ 20 Love2Shop Voucher to compensate for their time.

**Participant consent form**

Please complete this form after you have read the Information leaflet and listened to an explanation about the research.

**Title of Study:** Exploring Loneliness among people with ‘Complex Emotional Needs’ (CEN): A Qualitative Study

**Department:** Division of Psychiatry

**Name and Contact Details of the Researcher(s):** Sarah Ikhtabi, [sarah.ikhtabi.19@ucl.ac.uk](mailto:sarah.ikhtabi.19@ucl.ac.uk)

**Name and Contact Details of the Principal Researcher:** Sonia Johnson, [s.johnson@ucl.ac.uk](mailto:s.johnson@ucl.ac.uk)

**Name and Contact Details of the UCL Data Protection Officer:** Alexandra Potts [dataprotection@ucl.ac.uk](mailto:dataprotection@ucl.ac.uk)

This study has been approved by the UCL Research Ethics Committee: Project ID number: 23645/001

Thank you for considering taking part in this research. The person organising the research must explain the project to you before you agree to take part. If you have any questions arising from the Information leaflet or explanation already given to you, please ask the researcher before you decide whether to join in. You will be given a copy of this Consent Form to keep and refer to at any time.

I confirm that I understand that by ticking/initialling each box below, or verbally agreeing to the statement, I am consenting to this element of the study. I understand that it will be assumed that unticked/initialled boxes, or statements that I do not verbally agree to, means that I DO NOT consent to that part of the study. I understand that by not giving consent for any one element that I may be deemed ineligible for the study.

|  |  | Tick Box |
| --- | --- | --- |
| 1. | *I confirm that I have read and understood the Information Leaflet/Sheet for the above study. I have had an opportunity to consider the information and what will be expected of me. I have also had the opportunity to ask questions which have been answered to my satisfaction *and would like to take part in the one-on-one interview.* |  |
| 2. | *I understand that it is a requirement for my audio data to be recorded in order to take part in this study. |  |
| 3. | *I understand that I will be able to withdraw my data up to one month after my interview has taken place. |  |
| 4. | *I would like to receive a copy of the anonymised transcription for my interview:  Yes/No |  |
| 5. | *I consent to participate in the study. I understand that my personal information regarding my experiences of loneliness will be used for the purposes explained to me. I understand that according to data protection legislation, ‘public task’ will be the lawful basis for processing and ‘research purposes’ will be the lawful basis for processing special category data. |  |
| 6. | *I understand that all personal information will remain confidential and that all efforts will be made to ensure I cannot be identified. |  |
| 7. | *I understand that confidentiality will be respected unless there are legitimate reasons for this to be breached (i.e. I hear anything which makes me worried that you or someone else may be in danger). In this case, the researcher will inform you of the decision that might limit your confidentiality. |  |
| 8. | *I understand that the data gathered from me in this study will only be used for this project. |  |
| 9. | *I understand that my data gathered in this study will be stored anonymously and securely, and personal identifiers will be excluded, and the interview will be pseudonymised. It will not be possible to identify me in any publications. |  |
| 10. | *I understand that my anonymised information may be subject to review by responsible individuals from the University for monitoring and audit purposes. |  |
| 11. | *I understand that my participation is voluntary and that I am free to withdraw at any time without giving a reason. |  |
| 12. | *I understand that if I decide to withdraw, any personal data I have provided up to that point will be deleted, up to one month after the interview. |  |
| 13. | *I understand that there are limits to confidentiality associated with Microsoft Teams |  |
| 14. | *I understand the potential risks of participating and the support that will be available to me should I become distressed during the course of the research. |  |
| 15. | *I understand that there are no promises or guarantees of benefits that have been made to encourage me to participate. |  |
| 16. | *I understand that the data will not be made available to any commercial organisations but is solely the responsibility of the researcher(s) undertaking this study. |  |
| 17. | *I understand that I will not benefit financially from this study or from any possible outcome it may result in, in the future. |  |
| 18. | *I understand that the information I have submitted will be published as a report |  |
| 19. | * I wish to receive a copy of the published report    Yes/No |  |
| 20. | *I consent to my interview being audio recorded and understand that the recordings will be destroyed immediately *after the researcher conducting this study finishes her PhD, on October 1, 2025* |  |
| 21. | *I consent to my transcribed depersonalised data and audio recorded verbal consent and written consent form to be stored up to 10 years after the study. |  |
| 22. | *I hereby confirm that I understand the inclusion criteria as detailed in the Information Leaflet/Sheet and as explained to me by the researcher. |  |
| 23. | *I am aware of who I should contact if I wish to lodge a complaint. |  |
| 24. | *I voluntarily agree to take part in this study. |  |
| 25. | *I understand that other authenticated researchers involved in this study will have access to my anonymised and pseudonymised data. |  |

Name of participant

Date
