# Supplementary material for: Experiences of loneliness among people with a diagnosis of ‘personality disorder’: A qualitative interview study in the UK
Source: PLOS Ment Health. 2026 Aug 10;3(8):e0000671. doi: 10.1371/journal.pmen.0000671 (PMC13456265; doi:10.1371/journal.pmen.0000671)
Supplement: Supplementary file 4 [file pmen.0000671.s004.docx]

# **Appendix 4: Themes, Subthemes, and Illustrative Quotes**

| **Theme 1**  The experience of loneliness: 'personality disorder’, a lonely and disconnecting disorder    This theme reflected an intense sense of disconnection and enduring and pervasive feelings of loneliness. Loneliness was described as feeling persistently misunderstood, meaningless, faulty, and rejected, and a major feature and/or measure of ‘personality disorder’. | Sample of Quotes    *“You know on a regular basis I feel less human, I feel less a part of society, of my community. Umm, you know and the rest of humanity. And I'm not part of that, that wider group. And so I feel less alive and less vital. And it* [loneliness] *makes my whole existence feel less meaningful”*      *“I guess when I don't feel like understood by someone or. I don't feel like I can go to anyone for help”*    *“As a consequence of CEN* [‘personality disorder’]*, you know, it leads to other things, one of which is loneliness”*      *“It* [loneliness] *just makes me feel like a reject like ostracized. And people not understanding who I am”*      *“And I've certainly experienced, quite a lot of loneliness since I was a young child, I think and it, the feeling doesn't change over time. It feels like it's actually part of my DNA. So, despite being in a relationship, having a child, the state of loneliness feels like it's obviously come into my sense of identity at a sufficiently young age. It’s become a part of me”*    *“I've never been able to find that security that I want and to feel sort of kind of accepted and safe, so it almost loneliness almost feels, umm like a taint. Like there's something wrong with me, wrong with the way that my brain functions”*    *“See I just think there's such a close correlation for me between loneliness and my BPD and my mental health that it's hard to. What's the word? See the distinction. You know it's very … lines are very blurred”*      *“I know that with BPD we have issues with like identity and things, so like feeling different, feeling like we don't belong somewhere....I think, yeah, like lack of understanding of yourself and lack of being able to perceive yourself can make you feel very lonely”*      *“It's* [loneliness] *just feeling like there's just no one and nothing and sort of the feeling of like emptiness and I guess just being aware that there's other people or other things in existence, but you're just not able to connect”*  *“I think it just makes you feel a little bit disconnected sometimes. I think for me, it just makes me feel sometimes like that I can't belong. I can't belong in the world that I don't have a place sometimes”*      *“I feel great pain. No one to actually give me a listening ear, that understand my plight and my condition. I feel not wanted. I feel not belonging. I feel this sense of abnormality and it doesn't go well because I wanna be heard”*    *“My understanding of my own personality disorder. A lot of it grew out of that lack of connection and containment”* |
| --- | --- |
| **Subtheme 1**  Connection between specific symptoms of mental health/'personality disorder’ and loneliness    This subtheme demonstrated how symptoms of ‘personality disorder’ and mental health difficulties contributed to and exacerbated feelings of loneliness. | *“My attachment sort of, I tend to automatically get very strongly attached to certain people. And then that can then bring that stronger feeling of loneliness if that person then sort of detaches themselves from the situation, if that makes sense. And so I think it's actually just sort of attachment with people and yeah getting easily attached to people, most definitely impact my feelings of loneliness”*      *“So, it's* [splitting] *is like going from an idealised state and then going to a devaluing state so that I could like, split on my best friend and I could just hate him and then being like excessively angry and stuff like that.... Yeah. So, it's just difficult to like to connect with him I guess”*    *“I think fundamentally what loneliness is, is it's an emptiness and a lack of core identity that I can refer back to, whether that's because of… I mean I have values, but they're probably not... I sometimes worry that I don't have a philosophical call that I kind of tend to mimic circumstances or change my mind according to who I'm with. So, there's a kind of chronic instability in my identity, which I think then makes me not really believe that I'm a real person at all. There's a kind of sense of almost.. not existing, but a kind of solipsistic view of the world and then I think that sense of loneliness”*      *“I think you know a big part of that* [loneliness] *is about being let down, feeling not part of anything, Uh, you know, having low sort of self-esteem and thoughts about yourself”*  *“You have this distorted view of yourself, you know. Because of all the criticism I've had over the years, and it's made me very sort of lonely and alone and feeling crap basically”*      *“I think a lot of it* [loneliness] *has come down to me not being able to connect with my own emotions... Because I think I was never really brought up to connect with my emotions and we didn't really talk about feelings and stuff like that, and for me, that kind of would simultaneously kind of connect with the sense of worth...So I think in that sense then that made me feel lonely because I thought, like, my feelings didn't matter. But also it taught me to ignore my feelings and not be able to identify them, because I just kind of shut them down and, like, none of this was conscious”*    *“Yeah, deﬁnitely the dissociation I experience. Because sometimes I feel so disconnected from my body and the world and any sensations like it is quite hard to feel connected to anyone else when you do not feel connected to yourself... Like my body... my sensations are just numb”*  *“Like, I feel like a zombie sometimes. Yeah, like I am just watching things happen like I'm not an active participant and yeah, when I am struggling, I tend to withdraw I don't contact friends and I don't really want to see people which makes me feel worse, but I can't get out of that cycle”*      *I think there can be a link* (between ‘personality disorder and loneliness)*. We might…drive people or just cause people to be distant... because the behaviour towards them ... they might get more angry or annoyed... that causes.. unnecessary arguments with loved ones”* |
| **Subtheme 2**  Impact of loneliness on specific mental health/'personality disorder' symptoms    This subtheme illustrated that loneliness contributed to anxiety, depressive episodes, emotional dysregulation, suicidal ideation and self-harm. | *“I think on a psycho emotional level it* [loneliness] *makes me feel down. Umm. Somewhat depressed. Umm. And then also, both emotionally, psychologically somehow physically cold and weaker”*  *“I felt that perhaps I had nothing to contribute as a human being, you know, in a relational sense....Umm. And in my mind, I was making the inference, but that because I had no appeal, I probably had no human worth. So, I felt very cold and depressed and disconnected”*    *“On a sort of emotional level. Like, if I'm feeling lonely, then you know my moods gonna sort of fluctuate quite a lot. And if one minute I'm feeling lonely and then next minute I'm sort of....I don't know I am sort of socially benefiting from a situation then, you know, my mood sort of going to be a bit more unstable. I think just the feeling of being lonely, it does sort of impact my, you know, how I'm feeling emotionally, quite a lot”*      *“And I sometimes feel more anxious as well, if I feel like lonely. Because sometimes, especially if it's a room full of people and I feel like I'm different and things like that, I think I kind of feel anxious ... that I'm gonna be targeted again as, like, a kind of you're different and we know you're different”*  *“I think the loneliness more feeds into the depression and anxiety rather than like strictly BPD symptoms... Ohh well, the only one that it would is like the kind of thing I did until recently, like I was, I had self-harmed for like 10 years-ish. So definitely before like now I think that would have been a big thing. It's like the loneliness would have reinforced those kind of thoughts and feelings”*      *“In the past, I think it's* [loneliness] *led to quite severe suicidal ideation”*  *“Umm one other thing which was self-harming quite a lot as well, which meant I don't wanna see people because I was like then I have to hide it”* |

| **Theme 2**  The mutually reinforcing relationship between loneliness and mental health difficulties  This theme illustrated the cyclical and self-reinforcing relationship between loneliness and ‘personality disorder’/mental health symptoms in which loneliness exacerbates mental health symptoms which then further intensifies loneliness and thereon. | Sample of Quotes    *“Unfortunately, that created a perpetuating cycle because I felt, you know, a fear of rejection. It made me avoid or at least not take up opportunities for contact....” “I have lots of people I get on with and you know who I can be myself with. But I wouldn't. I'm not emotionally tied to because I know that would cause me emotional chaos and make me vulnerable to my own emotional self... I kind of like, keep myself at a distance... And that at times, yes, can feed into loneliness just to protect myself from myself”*      *“I think it was kind of self-perpetuating… the more lonely I've felt, the harder it was for me to figure out how to connect with people or to find the energy to kind of say “OK” to overcome your loneliness, you have to connect with people”. “But when you're lonely, you don't feel like connecting. You know you have to kind of...you're supposed to do the opposite of, you know, of what you're actually feeling like doing... So yeah, so I think there's, there's definitely that link between loneliness creating a sense of depression, of being unable to connect to other people, all those connections being shallow”*      *“But it's like a self-fulfilling prophecy because and again it's all, it's all linked because, you know, someone lets me down. I then say right on your bike, don't wanna know you, see you later. Then I feel lonely. Then I start, you know, going down with all my emotions and everything, then I isolate. And I'm back to square one where, you know, I haven't got anybody to spend time with”*      *“So, it kind of like I feel like a lot of our symptoms do make us feel alone and I don't know if that's because, like, we're programmed to try and protect ourselves because we're like hypervigilant. But it almost is too much, then we feel alone because we've done it too much. Like we push people away. So, then we are gonna feel lonely. So, it's kind of, yeah, I think it would be a a very core bit of BPD”*      *“I think it* (self-harm) *was definitely related to loneliness and just a complete inability to cope with any like sort of negative emotions. But I think it was quite cyclical. Like I'd be lonely, and I'd be like basically be unable to cope with anything and then I'd like start self-harming and then I'd not want to see people. So, then it would just sort of feed into the whole cycle”* |
| --- | --- |

| **Theme 3**  The perceived origins of loneliness: Alienating childhood experiences and memories    This theme demonstrated that the sense of loneliness and disconnection experienced were perceived to originate from invalidating and alienating childhood experiences and memories that were sometimes described as traumatising. | Sample of Quotes    *“Like I've kind of had quite a difficult upbringing and there's quite a lot of stress and I guess that trauma, when it comes to being abandoned by someone and like I always feel like someone is going to leave or you know they're gonna, like, go there and away and it sort of makes me like trust less I guess. And then I feel that it's like all these people are only gonna be temporary”*    *“I think just the difficulty in like understanding other people like...I have grown up in an abusive household. And so, my expectation is that I'm going to get hurt like any moment. It's very like unpredictable environment to be in. And so, I kind of just assume that people are gonna hurt me if I'm doing like, anything at all slightly wrong”*      *“I think also a lot of it* [loneliness] *relates back to my relationship with my* [X]. *He has always been like this* [critical] *and it hasn't eased off anytime, you know, throughout his life. I had hoped that, you know, as he got older, it would have been easier, but actually it seems to be getting worse. Ah, that has really affected and shaped me and made me how I am because you know, nothing was ever good enough for him. You know, I never uh, reach the bar. You know, I just always fell short”*  *“I think it's because* [reason for loneliness] *when I was younger I had like a very mixed relationship with like, well I still have a very mixed relationship with my parents... Umm, but at the time what like what happened. It wasn't like grossly serious, but it just was like it was quite isolating. So, it kind of ..they contributed to kind of helping me feel like I was very different to other people and that like I was a bad person and things like that. So, and that was since* [age] *as far as I can remember. So it's like it's probably that same kind of thing because those then became like my core beliefs that I was kind of like different and not someone that people wanted to hang around with, not someone that people liked”*      *“My loneliness, I think, goes a lot deeper… because at a younger age we weren't allowed to talk about things within the family. My parents were from that era where we don't talk about sex. We don't talk about anything”*    *“From ages* [X] *to* [X]...*I had a really close and abusive relationship.. Not romantic but it was not my parents either, but it was someone close to me, really close to me that was very emotionally abusive and it was something that happened every single day… that planted the belief that I’m not smart enough, I am boring.. and now whenever there is something that could relate to that.. umm, it kind of sends me into a spiral”*      *“I can define loneliness as growing up without my parents and growing up in a harsh environment”*    *“It* [loneliness] *comes with the confusion growing up with two parents that are very diﬀerent and the inconsistence of everyone in my life. Even* [parental figure], *he loved me, and this was a way to love and so it got very modelled in my head that actually harm and abuse was care”* |
| --- | --- |

| **Theme 4**  A unique form of stigma described as *“a dirty little secret”*    The unique stigma and negative connotations associated with ‘personality disorder’ contributed to an intense sense of exclusion and alienation from the self and society. The stigma of ‘personality disorder’ compounded with the stigma and discriminatory experiences associated with protected characteristics (e.g. sexuality) further exacerbated feelings of loneliness. | Sample of Quotes  *“So like loneliness. I think that the time when I feel the most lonely is sometimes when…I kind of feel like I'm not being understood or like I'm trying to explain something and people don't understand and sometimes that does happen when you try and explain like BPD because people don't really understand.. like it's really hard to explain what it's like without somebody like actually experiencing it...And there's a lot of stigma about it as well. And I'm like, we're actually not all like that. That's like a very like, if you wanna follow the stigma like there will be someone who meets the criteria for that stigma. But we're not all like that! Like a lot of us just function in like normal society and you will meet us, we are not all in life in like jail....So like I've found like the term personality disorder, people I think automatically are like ohh that's serious and that's something that's odd because like you know in the media and in books and in like TV shows and stuff...if you don't know anything about it, you don't know anyone with it, you don't do your own research and things like that, you would obviously only think of the stories that you hear like and sometimes like when things are reported like, oh, there's been like a murder or there's been like, harassment or something. The first thing that they'll say is like, ohh… he had a personality disorder. Ohh. She had. And it's almost like ohh! That explains why they were a bad person. And that's so unhelpful because it's like I am not actually… I'm still in control of my actions and still like I've still got a conscience and like I'm still able to do that, I'm not this like uncontrolled person that like could do anything at any time and like is completely like immoral and things like that. It just doesn't. That's not me. Umm. And I think that's kind of what contributes with like feeling alone because you… it doesn't matter it almost like sometimes I feel like it doesn't matter what I'm gonna say…the person that they think I am is possibly gonna win in their head because it's what they've learned about”*    *“So, I've liked dated girls and I'm like I'm pretty confident that it is who I am. It's very hard to be that within like a* (religious group)*, because it's frowned upon, you know, so it's kind of it's hard because I've not met anyone who's like me because we can't be public really about it. And I understand why and, it's OK. But it's hard because you, I don't know anyone who's feeling like me.... I think it kind of does make me feel a bit isolated because I'm not like... the struggles I'm having are not the same, and I can't talk about them either. So, it’s kind of, it does feel like isolating. I think in that sense”*      *“Yeah. I mean obviously, you know, I don't mind saying, obviously I have a PD diagnosis and I think there's that like misunderstanding of what that actually means for someone as in for wider society, the press, the media. I think you know, whenever it's sort of made public, you know, if ever it comes into the limelight, it's always presented in a negative way. And then the times that I have sort of disclosed it to work colleagues, it's been very misunderstood. You know almost causing concern for their safety and welfare and so then I think you become less likely to share that information again so it becomes like a dirty little secret. And sometimes I guess in like the way that you present yourselves you may, just be different. And so I think it kind of like self-perpetuates the fact that you have that and then it makes you feel that you can’t then fit in with like mainstream so that you do get that sense of exclusion and you are generally like on the fringes...I think there's like a misunderstanding of the diagnosis which just adds to the loneliness”*      *“I think the fact that I'm so sensitive in terms of personality is, you know, increased it* [loneliness]*. Like also umm like my sexuality and trying to see how that could possibly work together with my upbringing because I was brought up in a very* (religious) *family” ... “I started to kind of realize that I had feelings for women when I was about* [age]. *Umm but then I just kind of shut that down because it wasn't an option and it was over the next few years that you know that it kind of kept cropping up, but I kept on pushing it down. And that kind of made me feel, I think, more lonely and more isolated and more shame. And I think the shame increases loneliness massively, so yeah, there was a lot….I felt a lot of shame, a lot of internalised homophobia and that led me to suicide attempts and a lot of self harm, hospitalizations with my eating disorder and sort of have self harm and stuff like that and just felt very…yeah, very lonely with that and I didn't feel like I could tell anybody about it because I felt that I'd be judged”*      *“I haven't told really any of my friends that I have this label like it's kind of a secret. I'm way more comfortable saying, oh, I'm anxious or like my mood is low, because I think there's less stigma with that than with EUPD or borderline personality”*      *“I see myself as my gender orientation as not normal because you grow up in the society where people have a definition for normalcy and you are only left to define yourself as abnormal. And that led to some form of being lonely”*      *“I usually try meet groups. I used to try going to them but I found that it is so much work getting there and proving who I was that it just became more work than.. because of my* [disability] *they make a lot of assumptions about my intellect. I constantly have to prove myself”*      *“Yes, my sexuality and gender identity really played a big part of that disconnection I felt from myself. Um..throughout my life I found it very diﬃcult to connect with people because I wasn't showing my genuine self. Ohh, what's that word begins with the? My authentic self, I wasn't able to share my authentic self with people cause I'd buried so much of my authentic self as protection against more bullying… to hold on to the relationships I did have, even though they were painful and I was upset a lot of the time and was just agreeing to doing things with people just to have some people around me, so I didn't feel as lonely, but it was still a very lonely place to be in because I wasn't expressing what my needs were. And it got so buried I didn't even know what my needs actually were. And that's something I'm still untangling like what I actually want, what I actually need and how to express it”* |
| --- | --- |

| **Theme 5**  Recovery rooted in managing to find a sense of belonging: *“Once you feel heard, things start to change”*    This theme described the importance of a sense of belonging in promoting recovery and wellbeing. Participants reported using a combination of self-help, psychological or therapeutic approaches/skills alongside finding valued safe social spaces that are emotionally containing, accepting and based on commonality and mutuality to promote a sense of belonging. | Sample of Quotes  *“When I'm interacting with people, I feel so much happier, so much more... Umm, you know, I feel, you know, actualised. I feel that my life has a purpose and a meaning. And you know I'm useful in a more wider sense”*      *“I guess that when I was in a* (group space) *like there was people that were going through the same thing, then there was like, you know, that mutual connection between the people in the* (group) *and yourself so yeah, I do. I do think that like when I was on the* (group), *it was predominantly that positive relationships that I had and because you were going through the same thing”*      *“The thing what has helped me the most, which would be that being able to identify how you're feeling and being able to communicate that with the relevant person is the most helpful thing that I've ever done …because that basically that combination of having been able to connect with yourself and then being able to relay that to somebody who you can trust it is the most helpful thing and get to the point where you can do that…sometimes well, for me that required a lot of therapy”* |
| --- | --- |
| **Subtheme 1**  Finding a safe milieu for connecting to the social world    This subtheme illustrated that a sense of belonging is achieved within safe and containing social space that allow for authenticity, openness, and emotional exploration. These spaces could be therapeutic support groups, group activities/tasks, or/and healing and safe friendships. | *“So it was that sense of common purpose of we all, even though, we are very different, we were kind of all, we had that common goodwill towards each other. Ohh and you know and I guess again, although we had very different values. Umm we were all, you know, equally dedicated. So, you know we had an awful lot in common” “But of course we were different, bringing different things to the* [task], *so we were all learning from each other and yeah synergizing. So you know, it was, it was a, you know, really strong little unit to work with”*      *“When I am feeling lonely. I call him. It's almost like a… It's almost like a security blanket for me, so when I'm feeling very lonely and I just wanna talk to someone and someone that understands me, then I kind of like, speak to him” “it's definitely a safe space. It's definitely like I get him, he gets me. We both feel comfortable around each other”*      *“And then it was only like I think they* [the loneliness and feelings] *got less because…I moved …and I attended and I got involved in like my local* [group activity] *like I had been at home but down here I made friends and built relationships with people who were actually like, Umm, like good to me and like, didn't just treat me like I was like different or weird or anything like that”*      *“I have a job that keeps me well, if that’s the right phrase.. Keeps me connected, keeps me around people .. stops me from feeling lonely because I can relate to other people and.... I would just say find your tribe, you know, like find that thing where you feel connection and a lot of that is also going to come with, like mutuality and commonality. Umm, you know, for me that started through fitness. So, it's just kind of like finding that thing in that place. And just straight away I think just finding someone to listen and validate your experiences and feelings. Once you feel heard things start to change”*      *“I've joined a* *group that's like an LGBT* *group as well. So, I'm in this group and it's all people who are on the LGBT spectrum of some description and just get together. So, like, no right or wrong. It's just any thoughts or any opinion is valid…so it feels really relaxed and so that has been a massive thing for me having that community”* |
| **Subtheme 2**  Psychological strategies for coping with loneliness    This subtheme described a range of psychological strategies and coping tools used to alleviate loneliness. These range from self-help tools and activities such as the gym and nature, self-care and compassion techniques such as positive self-talk, and individual therapy and emotional processing and reflection. | *“So over the years I learned how to manage my emotions. Over the years I've got better at it because I found ways to be in contact with people which is safe for me. So.. I don't put myself in a vulnerable position umm...I feel relationships with others are better defined so I don't feel, you know, I'm not caught in the trap of feeling guilty and that I'm not doing enough for the person. Because I feel you know the boundaries of exchange, as it were, are much clearer”*    *“But like being a bit more like compassionate towards myself. So, for example, if I'm feeling abandoned or lonely Then I kind of go. I go to myself and like, OK, what I'm actually feeling and then like try to sort of self sooth myself.. So yeah, just being more compassionate towards my feelings and what I'm going through”*      *“I would say it* (walk in nature) *helped me with my loneliness because it makes you forget about your loneliness because you're outside”*      *“I'm just trying to connect with other people with my hobbies. I guess I don't really do that like in person. I just sort of like look online at people who do the same thing as me and to see other people be happy about it. And then I've got that connection there so that sort of thing”*  *“And I find when I'm engaged in something that is difficult but not impossible and that requires usually for me some kind of physical dexterity that engages me so much that all the sort of feelings of angst and loneliness and you know, wanting to drink, you know, it just kind of pushes all of those things out of the way”*      *“I think being able to be kind to yourself helps you to be kind... I was, I've always been kind to others, but I think being able to be compassionate to yourself allows others to be compassionate to you in a different way which I think increases that connection ..but also you’re able to like.. I was able to connect with myself more and that been a massive part of being able to connect with other people”*      *“I think exercise really helps like just if I'm feeling really low, not even just as like a tool to stop immediate self-harm, but just to like get outside and then just to sort of come back and feel like good about yourself, that you've moved, like the endorphins definitely helps”*    *“I am battling to keep so much in and contained, that was taking a lot of my energy and it was a big part of pushing people away... but I think sharing that with the therapist reduced what I was carrying and it allows new experiences to come in. and I think because she was the first person I told a lot of it to and she didn’t react in horror.. it made me think ohh! maybe other people can accept me. Maybe am not as bad as I have been told I am”* |
| **Subtheme 3**  Unhelpful ways of coping with loneliness    This subtheme described how some participants used unhelpful and temporary ways to cope with and numb the pain of loneliness. | *“I think you know I isolated myself to some extent and buried myself in my work to protect myself from what I feared would be rejection or being exposed as not being interesting” “You know my life was very mundane, it revolved around work and study, and I was not really alive”*      *“I guess like if I'm feeling more lonely, then I go to my eating disorder to get this sort of sense of control of or something like a sense of control. So like, say, if I feel more lonely then I might like restrict my food or, you know, do some sort of like eating disordered behaviour which obviously isn't good for me”*      *“Then also using alcohol, I sort of, you know, at a young age discovered that I would regulate better when I was drinking, as you know, there's a very distinct correlation between BPD and substance abuse” “And then I think that sense of loneliness, for me, the easiest thing I could do was drink to just…It's basically it was to numb the loneliness. That's exactly why I drank as much as I did, I suspect”*      *“The harm thing kind of put me back in control a little bit like… You know, you know, numb things or made you feel a lot. And so, it's kind of like dual purpose maybe in that sense. Umm, so yeah, I guess it's like control in there and just justifying the way I was feeling emotionally and ultimately like in relation to loneliness as well, I guess”*      *“You know, it would, by doing the self-harm it would actually relief me of that element of loneliness”*      *“So in the past I couldn't identify it* [feelings]. *I just felt like I knew that something wasn't right. Like I felt horrible, but I didn't know what it was like, to communicate it, and then often that would be when I would take things out on myself…* *you know end up with self-harming or just hiding away or my depression will get worse, and you know things like that”* |
