# Supplementary material for: Experiences of loneliness among people with a diagnosis of ‘personality disorder’: A qualitative interview study in the UK
Source: PLOS Ment Health. 2026 Aug 10;3(8):e0000671. doi: 10.1371/journal.pmen.0000671 (PMC13456265; doi:10.1371/journal.pmen.0000671)
Supplement: Supplementary file 5 [file pmen.0000671.s005.docx]

**Appendix 5:**

**Reflexivity and Areas of Discussion**

We provide below some examples of areas of discussion and challenges raised that prompted revision, revisiting interview transcripts, and re-organisation of themes:

- The primary author shared a preliminary thematic framework with the wider team after members of the team independently coded a proportion of transcripts and discussed emerging themes together. The team discussed the distinction between the way in which loneliness contributed to symptoms of ‘personality disorder’ and vice versa and how this is distinct from the mutually reinforcing relationship between loneliness and ‘personality disorder’. This prompted the primary researcher to refer back to interview transcripts to identify and disentangle the relationships between symptoms of ‘personality disorder’ and loneliness. We also decided to separate these subthemes on the relationship between loneliness and symptoms of ‘personality disorder’ (subtheme 1.1. and 1.2) from the theme on the mutually reinforcing relationship between loneliness and ‘personality disorder’ (theme 2).
- The researcher shared a draft of the first two themes with a qualitative research expert. The researcher initially included general descriptions of loneliness. Through discussion with a qualitative researcher, the primary researcher reviewed this and focused on ‘personality disorder’- specific experiences of loneliness.
- After sharing a draft of the themes with a qualitative research expert, a major area of discussion was exploring variability across each theme as opposed to focusing solely on commonalities across transcripts. The researcher revisited the interview transcripts and descriptive themes to identify differences across themes. For instance, the primary researcher referred back to interview transcripts and found that a few people did not report childhood trauma as a source of loneliness which was initially missed.
- After members of the team independently coded a proportion of transcripts, the team discussed the difficulties associated with distinguishing between whether loneliness preceded loneliness or vice versa. The researcher referred back to interview transcripts and descriptive themes to review this however we cannot ascertain whether loneliness preceded loneliness or vice versa.
- In the first draft of the result section, an area of discussion related to how to balance a narrative approach to capture participant’s experiences while also maintaining a clear thematic structure. This prompted the removal and addition of quotes/content. This was particularly relevant in relation to theme 3 on childhood experiences and memories.
- After the revision of the final draft, a discussion with a clinician specialising in the clinical care of patients with a diagnosis of ‘personality disorder’ highlighted i) the importance of dissociative experiences and a sense of instability in one’s identity as a feature of ‘personality disorder’ that is linked to loneliness and ii) how instability and disconnection within the self meant that people experienced difficulties connecting to others (theme 1, subtheme 1.1).
- Initially the primary researcher made a distinction between helpful and unhelpful ways of coping with loneliness however through revisions of drafts it became clear that we cannot clearly distinguish between whether skills were helpful or unhelpful as they may have served a purpose and can worsen/alleviate loneliness depending on circumstances. The primary researcher did notice that these skills that may be consider unhelpful – at times – were abandoned by older people.
- The researcher was prompted through feedback from the team to review themes and further explore the role of self-harm and its potential function as a way to manage loneliness and its potential impact on loneliness.
- The research team prompted the researcher to revisit themes and explore whether there are specific patterns by age (e.g. older vs younger) or type of trauma (emotional vs physical abuse).
- The team provided feedback on the importance of distinguishing between reported ways of coping with loneliness (e.g. distraction techniques) versus gaining a sense of belonging and directly addressing loneliness (e.g. joining social/group activities) (Theme 5) to avoid conflating the two.
